# Statin Use is Associated with a Less Severe Disease Course In Inflammatory Bowel Disease: A Nationwide Cohort Study 2006-2020

**DOI:** 10.1093/ibd/izaf077

**Published:** 2025-04-25

**Authors:** Hamed Khalili, Anders Forss, Jonas Söderling, Gabriella Bröms, Carl Eriksson, Jiangwei Sun, Jonas F Ludvigsson, Ola Olén

**Affiliations:** Clinical and Translation Epidemiology Unit, Massachusetts General Hospital and Harvard Medical School, Boston, MA, USA; Division of Gastroenterology, Department of Medicine, Massachusetts General Hospital, Boston, MA, USA; Clinical Epidemiology Division, Department of Medicine Solna, Karolinska Institutet, Stockholm, Sweden; Centre for Digestive Health, Department of Gastroenterology, Dermatovenereology and Rheumatology, Karolinska University Hospital, Stockholm, Sweden; Clinical Epidemiology Division, Department of Medicine Solna, Karolinska Institutet, Stockholm, Sweden; Clinical Epidemiology Division, Department of Medicine Solna, Karolinska Institutet, Stockholm, Sweden; Division of Gastroenterology, Department of Specialist Medicine, Danderyd Hospital, Stockholm, Sweden; Clinical Epidemiology Division, Department of Medicine Solna, Karolinska Institutet, Stockholm, Sweden; Department of Gastroenterology, Faculty of Medicine and Health, Örebro University, Örebro, Sweden; Department of Medical Epidemiology and Biostatistics, Karolinska Institutet, Stockholm, Sweden; Department of Medical Epidemiology and Biostatistics, Karolinska Institutet, Stockholm, Sweden; Department of Paediatrics, Örebro University Hospital, Örebro, Sweden; Division of Digestive and Liver Disease, Department of Medicine, Columbia University Medical Center, New York, NY, USA; Clinical Epidemiology Division, Department of Medicine Solna, Karolinska Institutet, Stockholm, Sweden; Sachs’ Children and Youth Hospital, Stockholm South General Hospital, Stockholm, Sweden

**Keywords:** Crohn’s disease, inflammatory bowel disease, statins, surgery, ulcerative colitis

## Abstract

**Background:**

Statins reduce the risk of inflammatory bowel disease (IBD), however their effect on IBD disease progression is largely unknown.

**Methods:**

We linked Swedish healthcare registers and performed a nationwide cohort study (2006-2020) of 19 788 adults (≥18 years) with ulcerative colitis (UC) and 12 582 with Crohn’s disease (CD). Of these, 1733 with UC and 962 with CD were identified as incident statin users after UC or CD diagnosis. After 1:1 propensity score matching, we compared statin users with non-users to estimate the risk of IBD-related surgery, hospitalizations, and disease flares expressed as incidence rates (IRs) and multivariable-adjusted hazard ratios (aHRs) with 95% confidence intervals (CIs). For outcomes with statistically significant estimates, we calculated the numbers needed to treat (NNT).

**Results:**

During a median follow-up of 3.4 years we observed a reduced risk of IBD-related surgery in statin users (UC, IR: 3.4 [95%CI: 2.1-4.8] per 1000 person-years; CD, IR: 9.2 [6.2-12.2]) compared with non-users in UC (IR: 6.3 [4.2-8.5]; aHR: 0.55 [0.31-0.97]) and CD (IR: 15.4 [11.0-19.7]; aHR: 0.54 [0.33-0.88]). The NNT to avoid one IBD-related surgical event per year of statin treatment were 345 (UC) and 161 (CD). For statin users, the risks of hospitalizations (IR: 17.0 [13.9-20.2]; aHR: 0.68 [0.51-0.91]) and disease flares (IR: 207.4 [193.2-221.6]; aHR: 0.86 [0.77-0.97]) were reduced in UC, but not in CD (IR: 20.3 [15.8-24.9]; aHR: 0.78 [0.56-1.09] and IR: 245.5 [223.9-267.1]; aHR: 1.02 [0.88-1.19]). In UC, NNT for hospitalizations and disease flares were 145 and 15.

**Conclusions:**

Statins were associated with a reduced risk of IBD-related surgery, hospitalizations, and disease flares in patients with UC, and with a reduced risk of IBD-related surgery in patients with CD.

**Key Messages**

**What is already known?**

Statins may reduce the risk of developing inflammatory bowel disease (IBD), but their impact on IBD progression remains unclear.

**What is new here?**

The use of statins is associated with a reduced risk of IBD-related surgery, hospitalizations, and disease flares.

**How can this study help patient care?**

Statins may potentially modify and reduce the risk of disease progression in patients with IBD.

## Introduction

Inflammatory bowel diseases (IBD), including Crohn’s disease (CD) and ulcerative colitis (UC), are chronic inflammatory conditions affecting the gastrointestinal tract. Globally, around 7 million individuals are impacted by IBD, leading to significant morbidity and economic burden on healthcare systems worldwide.^1^ Beyond genetics, dietary, and lifestyle factors, certain medications, such as non-steroidal anti-inflammatory drugs (NSAIDs) and oral contraceptives, have been identified as potential risk factors for IBD that may also be associated with disease progression and flares in people with established disease.^2,3^

Statins (hydroxymethylglutaryl-CoA reductase inhibitors) are among the most frequently prescribed drugs worldwide, with the use as primary and secondary prevention of cardiovascular diseases and treatment for hypercholesterolemia.^4^ Beyond that, statins exhibit pleiotropic effects, including modulation of inflammation, as evidenced by decreased levels of C-reactive protein (CRP), effects on cellular proliferation, inhibition of T-cell activation and leukocyte tissue infiltration, and induced apoptosis.^5–10^ Interestingly, recent research suggests that statins are associated with a reduced risk of incident IBD and reduced risk of colorectal cancer and overall mortality in prevalent IBD.^11–13^ Moreover, studies have reported associations between statins and proxies for advantageous disease progression such as reduced use of corticosteroids during IBD flares, decreased levels of inflammatory markers in IBD, and a reduced risk of colectomy.^14–17^ However, previous studies suffer from small sample sizes,^15,16^ limited generalizability,^17^ and use of surrogate markers of disease outcomes.^18^ Based on previous findings, we hypothesized that statin use may impact IBD disease progression. Leveraging data from nationwide Swedish administrative healthcare registers, which include over 30 000 adults with incident IBD from 2006 to 2019, we aimed to investigate the relationship between statin use and IBD progression defined as risk of IBD-related surgery, IBD-related hospitalizations, and disease flares.

## Materials and Methods

### Setting and Data Sources

Sweden provides universal and equal access to tax-funded public health care, including coverage of prescription medication, to all residents.^19^ This cohort study used data from Swedish administrative healthcare registers with nationwide coverage (eg, the National Patient Register [NPR], the Total Population Register [TPR], the Prescribed Drug Register [PDR], the Swedish Longitudinal, Integrated Database for Health Insurance and Labour Market Studies [LISA],^20^ and the Swedish quality register for IBD [SWIBREG]).^21,22^ For details, see Supplementary Materials, Supplementary eMethods). Data in these registers were cross-linked through the unique personal identity number assigned to all residents.^23^

### Study Population

We identified all adult (≥18 years) individuals with a record of ≥ 1 International Classification of Disease (ICD) code for IBD (CD and UC) in the NPR 2006-2019 (for definitions of IBD subtypes see Supplementary eMethods Table M1) and ≥ 1 IBD-related medications (defined in Supplementary Table M1).^24,25^ Date of diagnosis was set to the last date of either first-recorded IBD diagnosis or first-dispensed prescription of IBD-related medications. A previous validation of IBD diagnosis in the NPR showed a positive predictive value of 93% when requiring ≥ 2 ICD codes for IBD.^26^ We expect our current definition of IBD, requiring dispensed prescriptions of IBD medications as well, to have at least a similar positive predictive value. We excluded all individuals with IBD-related surgery prior to IBD diagnosis, migration within 1 year prior to IBD diagnosis, and no dispensed IBD drugs (Supplementary Table M3 for surgical procedures and ICD codes and Supplementary Tables S1a and S1b for exclusion criteria).

### Statin Exposure

Statin use was ascertained by records of dispensed prescriptions from the PDR by the Anatomical Therapeutic Chemical (ATC) classification system code for statins (includes lipophilic statins: simvastatin and atorvastatin; and hydrophilic statins: pravastatin and rosuvastatin) (Supplementary eMethods Table M1 for ATC codes). These are the most common types of statins available in Sweden.^27,28^ Start of statin exposure was defined as the date of dispensation for the first statin prescription reaching ≥ 30 cumulative defined daily doses (DDD, the standardized measure of average daily drug consumption as defined by the World Health Organization) between 1 July, 2006 and end of follow-up December 31, 2020). Index date for exposure was set to the date of the first-dispensed prescription of ≥ 30 cDDD for any statin and was analyzed in a “once exposed, always exposed” approach. To ensure we did not include prevalent statin users as exposed, we excluded individuals with any statin prescription within 12 months prior to IBD diagnosis and up to 6 months after diagnosis (Supplementary Tables S1a and S1b).

### Matching

We established the cohort using a two-step matching process (for details see Supplementary eMethods). In the initial direct matching step, we conducted a 1:1 exact matching for statin initiators and non-users based on IBD subtype (CD or UC), sex, age at IBD diagnosis (18-<60, and ≥60), calendar year of IBD diagnosis (2006–2010, 2011–2015, and 2016–2019), and level of education (≤12 and >12 years). Due to the uncertainty of diagnosis, we did not include patients with inflammatory bowel disease unclassified (IBD-U).^24,25^ The index date for statin users was the first dispensation of statin, and for non-users, it was any outpatient encounter or medication dispensation within 2 weeks of statin treatment in the matched statin user (see Supplementary Table M1 for ATC codes).

In the subsequent propensity score matching (step 2, performed using the PSMATCH procedure in SAS version 9.4), we matched statin users and non-statin users, separately for CD and UC, in a 1:1 ratio using a nearest-neighbor matching algorithm without replacement. The matching variables were selected based on their known or potential confounding effects on the relationship between IBD and the outcomes in our study. The propensity score that predicts the probability of statin use was derived from a logistic regression model adjusted for demographic and socioeconomic characteristics, comorbidities, co-medications, and disease characteristics up to and including the index date (further detailed in Supplementary eMethods and Supplementary Table M1). We assessed balance using the standardized mean difference, considering an imbalance when the standardized difference value exceeded ≤−0.1 or ≥0.1 (Table 1).

**Table 1.** Baseline characteristics of patients with Crohn’s disease and ulcerative colitis 2006-2019 with and without statin treatment (after matching)

Characteristic	UC^a^			CD^a^		
	**Statin users** **(*N* = 1,733)**	**Non-statin users** **(*N* = 1,733)**	**Standardized** **difference**	**Statin users** **(*N* = 962)**	**Non-statin users** **(*N* = 962)**	**Standardized** **difference**
Sex, no.^a^^,^^b^						
Women	782 (45.1%)	782 (45.1%)	0	486 (50.5%)	486 (50.5%)	0
Men	951 (54.9%)	951 (54.9%)	0	476 (49.5%)	476 (49.5%)	0
Age at IBD diagnosis						
Mean (SD)	59.8 (13.0)	59.8 (16.3)	0.002	58.1 (13.3)	58.5 (15.5)	−0.029
Median (IQR)	61.0 (51.9–69.0)	61.1 (49.0–71.8)		59.1 (49.4–67.3)	59.5 (48.2–69.3)	
*Categories, no.*						
18 y–<40 y	132 (7.6%)	223 (12.9%)	−0.174	92 (9.6%)	118 (12.3%)	−0.087
40 y–<50 y	246 (14.2%)	242 (14.0%)	0.007	164 (17.0%)	157 (16.3%)	0.020
50 y–<60 y	433 (25.0%)	346 (20.0%)	0.120	241 (25.1%)	222 (23.1%)	0.046
≥60 y	922 (53.2%)	922 (53.2%)	0	465 (48.3%)	465 (48.3%)	0
Age at index date^a^^,^^b^						
Mean (SD)	64.3 (12.5)	64.1 (15.8)	0.010	62.8 (12.8)	63.1 (15.0)	−0.023
Median (IQR)	65.4 (56.1–73.1)	65.6 (53.9–75.9)		64.1 (54.2–72.1)	64.4 (52.6–73.5)	
*Categories, no.*						
18 y–<40 y	62 (3.6%)	152 (8.8%)	−0.217	48 (5.0%)	72 (7.5%)	−0.103
40 y–<50 y	169 (9.8%)	182 (10.5%)	−0.025	107 (11.1%)	117 (12.2%)	−0.032
50 y–<60 y	359 (20.7%)	303 (17.5%)	0.082	222 (23.1%)	195 (20.3%)	0.068
≥60 y	1 143 (66.0%)	1 096 (63.2%)	0.057	585 (60.8%)	578 (60.1%)	0.015
Healthcare region^b^						
North	145 (8.4%)	133 (7.7%)	0.025	84 (8.7%)	97 (10.1%)	−0.046
Central	439 (25.3%)	446 (25.7%)	−0.009	224 (23.3%)	227 (23.6%)	−0.007
Stockholm	247 (14.3%)	275 (15.9%)	−0.045	194 (20.2%)	197 (20.5%)	−0.008
West	378 (21.8%)	362 (20.9%)	0.023	129 (13.4%)	119 (12.4%)	0.031
South-east	169 (9.8%)	167 (9.6%)	0.004	100 (10.4%)	95 (9.9%)	0.017
South	334 (19.3%)	328 (18.9%)	0.009	223 (23.2%)	221 (23.0%)	0.005
Missing	21 (1.2%)	22 (1.3%)	−0.005	8 (0.8%)	6 (0.6%)	0.024
Country of birth, no.^b^						
Nordic country	1 587 (91.6%)	1 584 (91.4%)	0.006	842 (87.5%)	851 (88.5%)	−0.029
Other European country	59 (3.4%)	79 (4.6%)	−0.059	46 (4.8%)	50 (5.2%)	−0.019
Other non-European country	87 (5.0%)	70 (4.0%)	0.047	74 (7.7%)	61 (6.3%)	0.053
Level of education, no.^a^^,^^b^						
≤9 y	485 (28.0%)	445 (25.7%)	0.052	277 (28.8%)	258 (26.8%)	0.044
10–12 y	861 (49.7%)	904 (52.2%)	−0.050	451 (46.9%)	469 (48.8%)	−0.037
>12 y	377 (21.8%)	377 (21.8%)	0	226 (23.5%)	226 (23.5%)	0
Missing	10 (0.6%)	7 (0.4%)	0.025	8 (0.8%)	9 (0.9%)	−0.011
Year of IBD diagnosis, no.^a^^,^^b^						
2006–2010	749 (43.2%)	749 (43.2%)	0	451 (46.9%)	451 (46.9%)	0
2011–2015	711 (41.0%)	711 (41.0%)	0	367 (38.1%)	367 (38.1%)	0
2016–2019	273 (15.8%)	273 (15.8%)	0	144 (15.0%)	144 (15.0%)	0
Year of index date^c^, no.						
2006–2010	148 (8.5%)	146 (8.4%)	0.004	80 (8.3%)	80 (8.3%)	0.000
2011–2015	535 (30.9%)	537 (31.0%)	−0.002	313 (32.5%)	313 (32.5%)	0.000
2016–2020	1 050 (60.6%)	1 050 (60.6%)	0.000	569 (59.1%)	569 (59.1%)	0.000
Montreal classification CD^b^						
L2				156 (16.2%)	147 (15.3%)	0.026
L1/L3/LX				806 (83.8%)	815 (84.7%)	−0.026
B1				814 (84.6%)	780 (81.1%)	0.094
B2/B3				148 (15.4%)	182 (18.9%)	−0.094
Perianal				62 (6.4%)	64 (6.7%)	−0.008
Montreal classification UC^b^						
E1/E2	606 (35.0%)	606 (35.0%)	0.000			
E3	368 (21.2%)	428 (24.7%)	−0.082			
EX	756 (43.6%)	697 (40.2%)	0.069			
Missing	3 (0.2%)	2 (0.1%)	0.015			
Extraintestinal manifestations (EIM)						
Primary sclerosing cholangitis	23 (1.3%)	52 (3.0%)	−0.115	13 (1.4%)	23 (2.4%)	−0.077
Other EIM	316 (18.2%)	367 (21.2%)	−0.074	240 (24.9%)	251 (26.1%)	−0.026
Comorbidities ever before index^b^^,^^c^ date, no						
Diabetes	496 (28.6%)	409 (23.6%)	0.114	266 (27.7%)	227 (23.6%)	0.093
Hypertension	1 249 (72.1%)	1 368 (78.9%)	−0.160	691 (71.8%)	744 (77.3%)	−0.127
Dyslipidemia	1 733 (100.0%)	406 (23.4%)	2.557	962 (100%)	298 (31.0%)	2.111
Cardiovascular disease	991 (57.2%)	1 013 (58.5%)	−0.026	552 (57.4%)	538 (55.9%)	0.029
Medications ever before index date^c^, no						
Metformin use	360 (20.8%)	277 (16.0%)	0.124	183 (19.0%)	159 (16.5%)	0.065
Insulin use	197 (11.4%)	159 (9.2%)	0.072	110 (11.4%)	89 (9.3%)	0.072
Aspirin use	728 (42.0%)	614 (35.4%)	0.135	420 (43.7%)	364 (37.8%)	0.119
ACE inhibitor use	1 037 (59.8%)	1 101 (63.5%)	−0.076	554 (57.6%)	566 (58.8%)	−0.025
Date of IBD diagnosis, no.						
UC or CD diagnosis from NPR	739 (42.6%)	744 (42.9%)	−0.006	410 (42.6%)	443 (46.0%)	−0.069
IBD drug from PDR	994 (57.4%)	989 (57.1%)	0.006	552 (57.4%)	519 (54.0%)	0.069
Systemic steroid use	963 (55.6%)	962 (55.5%)	0.001	510 (53.0%)	482 (50.1%)	0.058
Immunosuppressive medication	23 (1.3%)	16 (0.9%)	0.038	36 (3.7%)	30 (3.1%)	0.034
Anti-TNF	8 (0.5%)	11 (0.6%)	−0.023	6 (0.6%)	7 (0.7%)	−0.013
Outcome ≤ 6 months of index date^c^						
IBD-related hospitalization	25 (1.4%)	42 (2.4%)	−0.071	18 (1.9%)	30 (3.1%)	−0.080
Systemic steroid use	442 (25.5%)	507 (29.3%)	−0.084	291 (30.2%)	302 (31.4%)	−0.025
Immunosuppressive medication	201 (11.6%)	209 (12.1%)	−0.014	144 (15.0%)	161 (16.7%)	−0.048
Started anti-TNF	17 (1.0%)	18 (1.0%)	−0.006	18 (1.9%)	24 (2.5%)	−0.043
Healthcare utilization ≤ 1 year before index date^b^^,^^c^						
*Number of outpatient care visits*						
Mean (SD)	4.0 (4.4)	4.2 (5.1)	−0.042	4.9 (7.9)	5.1 (7.9)	−0.031
Median (IQR)	3 (1–5)	3 (1–6)		3 (1–6)	3 (1–7)	
*Number of hospitalizations*						
Mean (SD)	0.8 (1.4)	1.0 (2.0)	−0.061	1.1 (1.8)	1.0 (2.6)	0.019
Median (IQR)	0 (0–1)	0 (0–1)		0 (0–1)	0 (0–1)	
*Number of prescription medications*						
Mean (SD)	35.5 (42.7)	34.7 (35.8)	0.020	36.2 (33.6)	34.7 (35.8)	0.044
Median (IQR)	26 (16–40)	26 (16–42)		28 (16–44)	27 (16–42)	
Charlson Comorbidity Index						
Mean (SD)	1.5 (1.8)	1.6 (1.9)	−0.028	1.5 (1.7)	1.6 (1.9)	−0.018
Median (IQR)	1 (0–2)	1 (0–2)		1 (0–2)	1 (0–2)	
*Categories, no.*						
0	633 (36.5%)	720 (41.5%)	−0.103	319 (33.2%)	368 (38.3%)	−0.106
1	411 (23.7%)	323 (18.6%)	0.125	251 (26.1%)	195 (20.3%)	0.138
2	319 (18.4%)	268 (15.5%)	0.079	171 (17.8%)	178 (18.5%)	−0.019
≥3	370 (21.4%)	422 (24.4%)	−0.072	221 (23.0%)	221 (23.0%)	0.000
Statin at treatment start, no.						
Simvastatin	567 (32.7%)			329 (34.2%)		
Atorvastatin	1 080 (62.3%)			589 (61.2%)		
Rosuvastatin	79 (4.6%)			37 (3.8%)		
Pravastatin	7 (0.4%)			7 (0.7%)		
Disease duration at index date^b^^,^^c^						
Mean (SD)	4.5 (3.1)	4.3 (3.0)	0.040	4.7 (3.3)	4.6 (3.2)	0.031
Median (IQR)	3.8 (1.8–6.4)	3.8 (1.7–6.4)		4.0 (1.8–7.0)	3.9 (1.9–6.7)	
*Categories, no.*						
<1 y	168 (9.7%)	187 (10.8%)	−0.036	108 (11.2%)	105 (10.9%)	0.010
1–<5 y	906 (52.3%)	932 (53.8%)	−0.030	481 (50.0%)	487 (50.6%)	−0.012
5–<10 y	544 (31.4%)	522 (30.1%)	0.028	296 (30.8%)	295 (30.7%)	0.002
≥10 y	115 (6.6%)	92 (5.3%)	0.056	77 (8.0%)	75 (7.8%)	0.008
Follow-up from index date to death, migration start of ≥ 30 consecutive cDDDs or end of data, years						
Mean (SD)	4.1 (3.2)	3.2 (2.7)		4.2 (3.2)	3.4 (2.9)	
Median (IQR)	3.4 (1.4–6.1)	2.4 (1.0–4.7)		3.4 (1.4–6.3)	2.6 (1.1–5.0)	
*Categories, no.*						
<1 y	302 (17.4%)	427 (24.6%)		174 (18.1%)	218 (22.7%)	
1–<5 y	855 (49.3%)	915 (52.8%)		443 (46.0%)	499 (51.9%)	
5–<10 y	463 (26.7%)	340 (19.6%)		283 (29.4%)	208 (21.6%)	
≥10 y	113 (6.5%)	51 (2.9%)		62 (6.4%)	37 (3.8%)	
*Reason for end of follow-up, no.*						
Death	198 (11.4%)	261 (15.1%)		118 (12.3%)	132 (13.7%)	
Emigration	11 (0.6%)	9 (0.5%)		5 (0.5%)	7 (0.7%)	
Start of statin ≥ 30 consecutive DDDs	–	313 (18.1%)		–	158 (16.4%)	
End of data (Dec 31, 2020)	1 524 (87.9%)	1 150 (66.4%)		839 (87.2%)	665 (69.1%)	

ACE inhibitor, angiotensin-converting-enzyme inhibitor; anti-TNF; anti-tumor necrosis factor alpha; CD, Crohn’s disease; cDDD, cumulative defined daily dose; DDD, defined daily dose; IBD, inflammatory bowel disease, IQR, interquartile range; NPR; national patient register; PDR; prescribed drug register; SD, standard deviation; UC, ulcerative colitis; y, years.

^a^This variable was used in the direct matching (for details see Supplementary eMethods).

^b^This variable was used in the propensity score matching (for details see Supplementary eMethods).

^c^Index date: >6 months after IBD diagnosis and start of ≥ 30 consecutive cumulative DDDs in statin users with UC and CD.

### Outcomes and Follow-Up

We studied three separate outcomes by IBD subtype (CD and UC): (1) incident IBD-related surgery (includes bowel resections and related stoma formation); (2) IBD-related hospitalization (defined as any hospitalization with a main diagnosis of UC or CD); and (3) disease flare (defined as any of systemic corticosteroid prescription, start of immunomodulator, or start/switch to anti-tumor necrosis factor (TNF) treatment). These outcomes were ascertained from data in the NPR and PDR (Supplementary Table M1 for surgical procedure codes, ICD codes, and medication ACT codes). Follow-up began at the index date and continued until the time of the first-recorded event (outcomes 1-3), emigration, death, or end of follow-up on December 31, 2020. If matched statin non-user comparators initiated statin treatment after start of follow-up, they stopped contributing follow-up time as a comparator (censored) and started contributing follow-up time as statin exposed patient instead.

### Statistics

We estimated incidence rates with 95% confidence intervals (CIs) for each of the outcomes (IBD-related surgery, hospitalization, and disease flare) separately for CD and UC comparing statin users with non-users. A patient could contribute with person-time to one of the three outcomes regardless of a prior incident event of any of the other outcomes. Conditioned on matching set, we used stratified Cox proportional hazards model to estimate adjusted hazard ratios (aHRs) with 95% CIs comparing statin users with non-users. We also calculated numbers needed to treat (NNT) for outcomes with aHRs that reached statistical significance. NNT calculations were based on the incidence rates (see Supplementary eMethods for description of calculations). Cumulative incidence for each outcome was presented as Kaplan–Meier plot. We performed subgroup analyses to investigate if potential associations between statin use and our outcomes varied (ie, signs of effect modification) by follow-up time, sex, age at IBD diagnosis (18-<60, and ≥60), calendar year of IBD diagnosis (2006–2010, 2011–2015, and 2016–2019), calendar year of start of follow-up, level of education (≤12 and >12 years), and type of statins (simvastatin, atorvastatin, or other statins). The proportional hazards assumption was assessed through Schoenfeld residuals related to time and was not violated. Statistical analyses were performed using SAS version 9.4 (SAS Institute Inc., Cary, NC). Two-sided *P*-values of ≤ .05 were considered statistically significant.

## Results

Between January 1, 2006 and December 31, 2019, we identified 19 788 incident UC and 12 582 incident CD patients. Among these patients, 1733 with UC and 962 with CD initiated statin therapy >6 months after IBD diagnosis. At the start of follow-up (and after matching), the median age for statin users with UC was 61 years (interquartile range (IQR) 52-69), and for CD was 59 years (IQR 49-67). In UC, most statin users were male (54.9%), while in CD the proportion was even (male 49.5% vs female 50.5%). The median follow-up for patients with UC was 3.4 years (IQR 1.4-6.1) and 3.4 years (IQR 1.4-6.3) for CD. Additional baseline characteristics are presented in Table 1 (after matching and in Supplementary Table S1 before matching). The baseline characteristics of participants were similar between statin initiators and non-users (Table 1).

### Statin Use and Risk of IBD-Related Surgery

**Figure 1.**
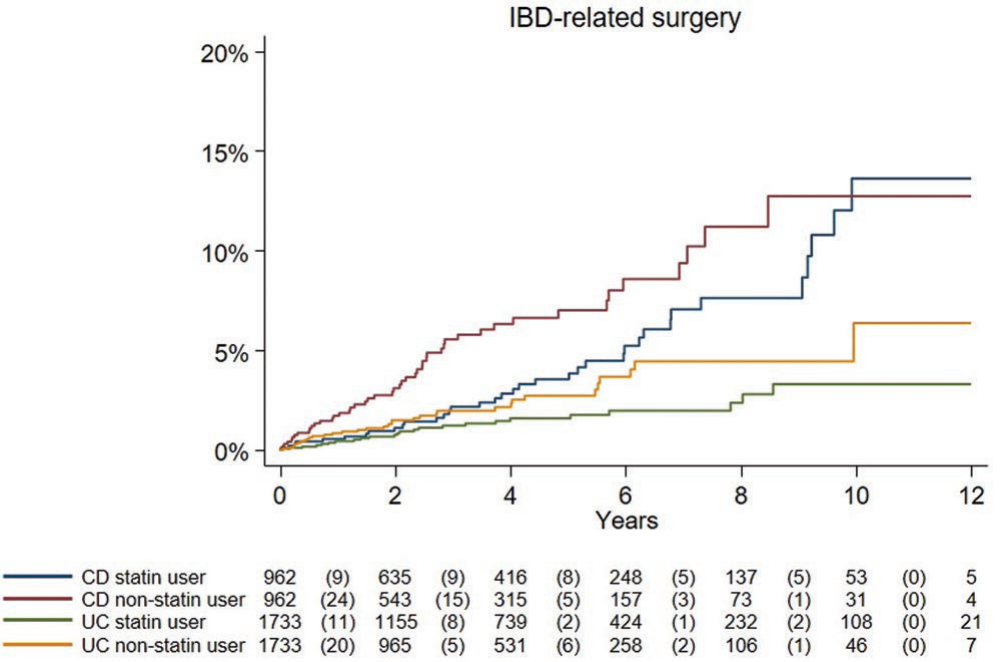
Kaplan–Meier curves (unadjusted) showing time from index date (statin initiation) to any inflammatory bowel disease (IBD)-related surgical event in patients with ulcerative colitis (UC) and Crohn’s disease (CD) comparing statin users and non-users. Number of events is shown in the parenthesis. (Log-rank test ulcerative colitis, *P* = .04; Crohn’s disease, *P* = .01).

In UC, we recorded 24 IBD-related surgical events (IR 3.4/1000 PY, 95%CI = 2.1-4.8) among initiators of statins as compared with 34 events in non-users (median follow-up 2.4 years [IQR 1.0-4.7]; IR 6.3/1000 PY, 95%CI = 4.2-8.5). This translated into an aHR of 0.55, 95%CI = 0.31-0.97 (Table 2, Supplementary Table S3a, and Figure 1).

**Table 2.** Incidence rates, hazard ratios, and number needed to treat of inflammatory bowel disease related surgery, hospitalization, and disease flare in patients with ulcerative colitis and Crohn’s disease with and without statin treatment 2006-2020.

Outcome	*N*		*N* events		Incidence rate (95% CI) per 1000 PY		HR^a^ (95%CI)	aHR^b^ (95%CI)	NNT per 1 year of statin treatment
	**Statin users**	**Non-statin users**	**Statin users**	**Non-statin users**	**Statin users**	**Non-statin users**			
IBD-related surgery									
UC	1 733	1 733	24 (1.4%)	34 (2.0%)	3.4 (2.1–4.8)	6.3 (4.2–8.5)	0.56 (0.33–0.95)	0.55 (0.31–0.97)	345
CD	962	962	36 (3.7%)	48 (5.0%)	9.2 (6.2–12.2)	15.4 (11.0–19.7)	0.59 (0.39–0.90)	0.54 (0.33–0.88)	161
IBD-hospitalization									
UC	1 733	1 733	113 (6.5%)	121 (7.0%)	17.0 (13.9–20.2)	23.9 (19.6–28.1)	0.77 (0.59–0.99)	0.68 (0.51–0.91)	145
CD	962	962	76 (7.9%)	84 (8.7%)	20.3 (15.8-24.9)	28.2 (22.2–34.2)	0.78 (0.57–1.06)	0.78 (0.56–1.09)	–
Disease flare									
UC	1 733	1 733	819 (47.3%)	825 (47.6%)	207.4 (193.2–221.6)	275.0 (256.2–293.8)	0.85 (0.77–0.94)	0.86 (0.77–0.97)	15
CD	962	962	496 (51.6%)	478 (49.7%)	245.5 (223.9–267.1)	292.3 (266.1–318.5)	0.96 (0.85–1.08)	1.02 (0.88–1.19)	–

aHR, adjusted hazard ratio; CD, Crohn’s disease; CI, confidence interval; HR, hazard ratio; IBD, inflammatory bowel disease; NNT, number needed to treat; PY, person-year; UC, ulcerative colitis.

^a^Propensity score matched for sex, age, level of education, year of diagnosis, disease duration, location and extent, healthcare utilization, and comorbidities.

^b^Conditioned on matching set.

In CD, we identified 36 IBD-related surgical events (IR 9.2/1000 PY, 95%CI = 6.2-12.2) among 962 statin initiators as compared with 48 events in non-statin users (median follow-up 2.6 years [IQR 1.1-5.0]; IR 15.4/1000 PY, 95%CI = 11.0-19.7). After multivariable adjustment, statin initiators were at 46% lower risk of IBD-related surgery than non-statin users (aHR: 0.54, 95%CI = 0.33-0.88) (Table 2, Supplementary Table S3b, and Figure 1).

In stratified analyses of IBD-related surgical events, no significant differences were found according to sex, age groups (18-<60 vs ≥60 years), year of diagnosis, length of follow-up (<5 vs ≥5 years), level of education, and period of follow-up in both UC and CD groups (Supplementary Tables S3a and S3b).

### Statin Use and Risk of IBD-Related Hospitalizations

**Figure 2.**
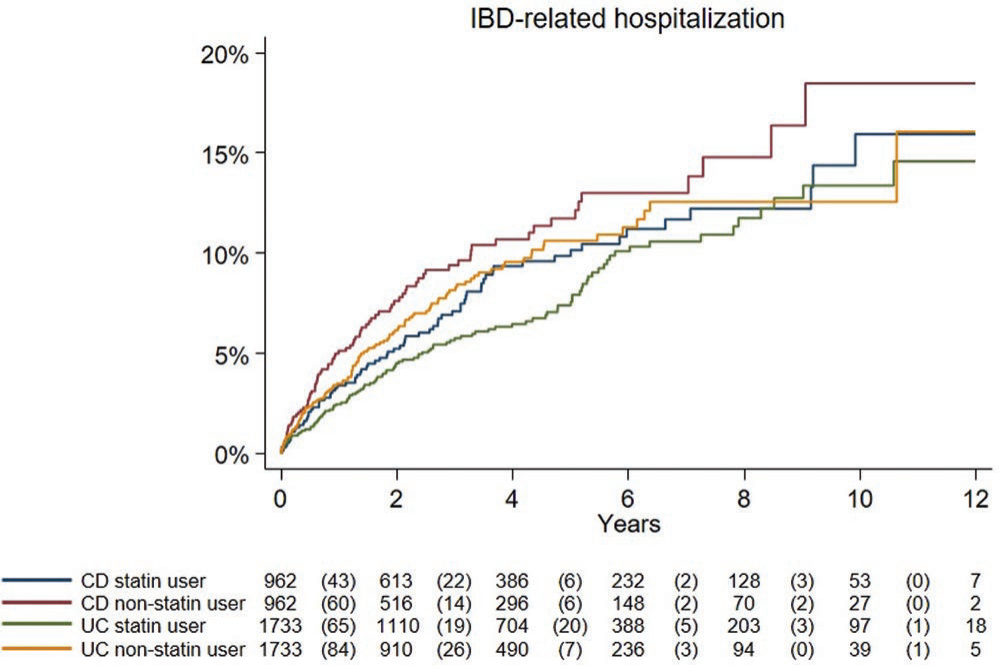
Kaplan–Meier curves (unadjusted) showing time from index date (statin initiation) to any inflammatory bowel disease (IBD)-related hospitalization in patients with ulcerative colitis (UC) and Crohn’s disease (CD) comparing statin users and non-users. Number of events is shown in the parenthesis. (Log-rank test ulcerative colitis, *P* = .01; Crohn’s disease, *P* = .15).

In UC, 113 IBD-related hospitalizations (IR 17.0/1000 PY, 95%CI = 13.9-20.2) were observed among statin initiators and 121 events in matched non-users (IR 23.9/1000 PY, 95%CI = 19.6-28.1). Statin initiation was associated with a 32% reduction in IBD-related hospitalization (aHR: 0.68, 95%CI = 0.51-0.91) (Table 2, Supplementary Table S4a, and Figure 2).

In CD, we recorded 76 IBD-related hospitalizations (IR 20.3/1000 PY, 95%CI = 15.8-24.9) among 962 statin initiators as compared to 84 IBD-related hospitalizations in matched non-users (IR 28.2/1000 PY, 95%CI = 22.2-34.2). Statin initiation was associated with a 22% reduction in risk of hospitalization, albeit the reduction was not statistically significant (aHR: 0.78, 95%CI = 0.56-1.09) (Table 2, Supplementary Table S4b, and Figure 2).

There were no significant differences according to sex, age groups, year of diagnosis, length of follow-up, level of education, and period of follow-up neither for patients with UC nor CD (Supplementary Tables S4a and S4b). Stratified by type of statin, only patients with UC treated with atorvastatin had a statistically significant reduced risk (aHR: 0.60, 95%CI = 0.40-0.90) of hospitalization.

### Statin Use and Risk of Disease Flare

**Figure 3.**
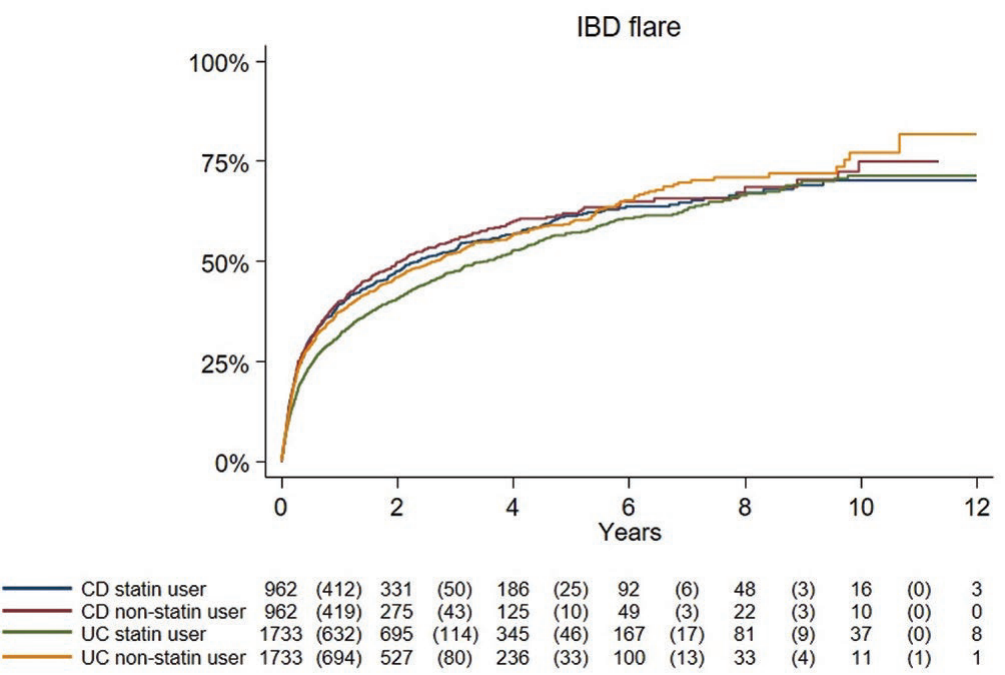
Kaplan–Meier curves (unadjusted) showing time from index date (statin initiation) to any inflammatory bowel disease (IBD)-related disease flare in patients with ulcerative colitis (UC) and Crohn’s disease (CD) comparing statin users and non-users. Number of events is shown in the parenthesis. (Log-rank test ulcerative colitis, *P* = .01; Crohn’s disease, *P* = .79).

In UC, we recorded 819 disease flares (IR 207.4/1000 PY, 95%CI = 193.2-221.6) among statin initiators compared with 825 flares in matched non-statin users (IR 275.0/1000 PY, 95%CI = 256.2-293.8) (Figure 3). Statin initiators were at a 14% lower risk of disease flares compared with non-users (aHR: 0.86, 95%CI = 0.77-0.97) (Table 2, Supplementary Table S5a, and Figure 3).

In CD, we observed 496 disease flares (IR 245.5/1000 PY, 95%CI = 223.9-267.1) in statin initiators compared with 478 events in non-users (IR 292.3/1000 PY, 95%CI = 266.1-318.5). Statin initiators had the same risk as non-users after multivariable adjustment (aHR: 1.02, 95%CI = 0.88-1.19) (Table 2, Supplementary Table S5b, and Figure 3).

Results were comparable, with overlapping confidence intervals, for the risk stratified by sex, age group, year of diagnosis, length of follow-up, period of follow-up, level of education, and statin type for any of the subtypes of IBD (Supplementary Tables S5a and S5b).

As a measure of absolute risk (differences), we calculated NNT for outcomes that demonstrated statistically significant associations with statin use. NNT to avoid one IBD-related surgical event per year of statin treatment were 345 patients for UC and 161 for CD. The corresponding NNT for hospitalizations and disease flares were 145 and 15 per 1 year of statin treatment for UC (Table 2, see Supplementary eMethods for description of calculations of NNT).

## Discussion

In this nationwide cohort of adults with incident IBD 2006-2019 (followed until the end of 2020) we found that the use of statins was associated with a reduced risk of IBD-related surgery, hospitalizations, and disease flares in patients with UC, while in CD, only a reduced risk of surgery was observed. Similar associations were observed after stratification by sex, age group, year of diagnosis, and follow-up period, although the results, likely due to lower statistical power, did not remain statistically significant for some comparisons.

Register-based cohort studies suggest a protective effect of statins against incident IBD.^11,12^ Therefore, statin use may constitute a preventive strategy in individuals identified at high risk of developing IBD. Statins have also been suggested to reduce all-cause mortality (aHR = 0.63, 95%CI: 0.57-0.69) in patients with prevalent IBD.^13^ However, only few studies have addressed the potential disease-modifying effects and the impact on disease activity of statins in individuals with prevalent IBD. In a US register-based cohort study by Crockett et al., statin use was associated with a 25% reduction in the risk of corticosteroid use (aHR = 0.75, 95%CI: 0.62–0.91) in patients with UC (*n* = 1132), but not with CD (*n* = 824; aHR = 0.91, 95%CI: 0.74-1.12).^14^ In their study, only atorvastatin showed a statistically significant reduced risk (aHR = 0.76, 95%CI: 0.60–0.96). These findings are largely consistent with our results. However, since the mentioned study was limited to individuals with IBD aged 35-64 years, while the majority of statin users initiate treatment at ages over 60 years. Moreover, the largest magnitude of the effect of statins in our study was seen in those aged 60 years and older. Our study, which was nationwide and included an unselected IBD population of adults ≥ 18 years with exact date on year of IBD disease onset, therefore, significantly expands on prior work. Furthermore, we applied different methodological approaches. We used propensity score matching, while Crockett et al. matched randomly sampled non-statin users with IBD using an incidence density design, matched on age and sex, and did not comprehensively account for several intraindividual differences that could bias the comparison between statin users and non-users. Applying a propensity score matching is likely to better account for such differences.

The association of statins with disease activity in IBD has also been investigated in a randomized controlled trial (RCT) (published as an abstract) where 36 patients with IBD received 40 mg atorvastatin and the Seo disease activity index score was assessed after 24 weeks of treatment.^18^ Patients treated with atorvastatin showed a borderline significant lower activity index score compared with the placebo group (132.1 vs 157.6, *P* = .06) at the end of the study period, but when adjusting for disease duration, atorvastatin treatment was associated with a significant decreased disease activity (*P* = .02). However, another RCT investigating the efficacy of atorvastatin in patients with UC and mild to moderately severe acute disease activity, measured as reduction of partial Mayo Score, did not detect any change in disease activity after 8 weeks of treatment.^29^ In fact, almost half of the atorvastatin-treated patients showed an increased partial Mayo Score (of ≥2 points), while none of the placebo-treated group showed an increase (*P* < .001). In two clinical trials where 10 CD patients were treated with 80 mg atorvastatin per day for 13 weeks, statin treatment was associated with a reduction in CRP and C-X-C motif chemokine ligand 10 (CXCL10, a cytokine attributed to several roles in the chemoattraction of different immune cells) levels (*P* = .008 and *P* = .026, respectively).^15,16^ However, these clinical studies were small, lacked comparison groups, and partly used surrogate markers for disease activity that are clinically less meaningful than the outcomes used in our study. Collectively, despite limitations of previous studies, current evidence suggests that statins potentially reduce levels of inflammatory markers and disease activity in patients with IBD.

To our knowledge, no RCT or large nationwide observational cohort study based on prospectively collected data has investigated rates of IBD-related surgery and hospitalizations in patients with IBD comparing statin users with non-users. However, Bai et al. investigated the risk of colectomy and time to first hospitalization in patients with UC exposed to atorvastatin compared with UC patients exposed to other cardiovascular and lipomodulatory drugs in two cohorts based on data from administrative healthcare databases.^17^ They found that atorvastatin reduced the risk of both colectomy and time to first hospitalization after treatment initiation even when adjusting for IBD therapy. Their findings are consistent with the reduced risk of IBD-related surgery and hospitalization in UC observed in our cohort. The generalizability of their findings is somewhat limited due to the selected study population sourced from insurance claims databases.

Possible mechanisms potentiating the reduced risk of IBD-related outcomes seen in our study include reduced disease activity and inflammation as statins have been shown to decrease systemic inflammation as measured by circulating CRP.^16^ Although only evidenced in animal models of dextran sulfate sodium (DSS)-induced colitis, statins induced a decrease in the expression of pro-inflammatory markers including TNF-alpha.^30^ Possibly through similar pathways, in 2,4,6-trinitrobenzenessulfonic acid (TNBS)-induced colitis, simvastatin decreased colonic inflammation and also exerted anti-fibrotic effects measured as a dose-dependent decrease in the level of a fibrosis-related growth factor.^31^ Moreover, in peripheral blood mononuclear cells isolated from healthy volunteers, treatment with statins increased the number of a set of T cells that play a key role in the development and maintenance of immune tolerance.^9,10^

We report negative associations for all outcomes in patients with UC, but only for some in CD. Similar differences were also seen in the study by Crockett et al.^14^ We have previously reported an association between statin use and a lower risk of incident CD, but not of UC.^11^ Speculatively, this could indicate different effects of statins between subtypes of IBD and in CD and UC before and after disease onset. Although, it cannot be excluded that seemingly contradictory findings of the effect of statins between incident and prevalent cases are due to insufficient sample sizes (the 95%CIs for aHRs of flares in CD and UC overlapped vastly, although only the estimate for UC was statistically significant [Table 2]).^11^ What makes a differential statin effect on UC and CD somewhat plausible, is that CD and UC are to some extent biologically heterogenous conditions for which differential associations with both exposures and outcomes have been reported. Differences in the abundance of microbial species have been found between patients with UC and CD.^32^ Statins have been suggested to alter the intestinal microbiota composition.^33–35^ Such statin-induced alterations could potentially modify the disease course of UC and CD differently, which could partly explain the differences in associations between the IBD subtypes. We posit that the association with statins and reduced complications in UC, but only surgery (and possibly hospitalizations, albeit not statistically significant) in CD, observed in our study does not preclude that these observations may reflect causal links. Further large cohort studies are needed to disentangle the seemingly contradictory effects of statins in CD and UC.

When we explored the risk by statin type, the results were inconsistent across the different outcomes and subtypes of IBD. However, atorvastatin was more frequently used and showed a statistically significant reduced risk of surgery in CD and of hospitalization in UC, whereas simvastatin only significantly reduced the risk of surgery in UC. Considering the inconsistency of our findings, we were not able to draw any conclusion about potential differential effects on IBD progression by statin type.

Our study has several strengths, such as the nationwide study population leveraging Swedish registers with nearly complete coverage and virtually no loss to follow-up, thus reducing the risk of selection and information bias.^26,36,37^ We also performed analyses separately for UC and CD, and analyses stratified by potential effect modifiers (eg, sex, age, education).

Our study also has potential limitations. First, as in all observational studies, we acknowledge the inherent vulnerability to residual confounding. Despite our efforts to limit confounding through propensity score-matched comparison and adjustment for several clinically relevant covariates, residual confounding could potentially have impacted our results. We speculate that the lack of data on lifestyle, diet, body mass index, and smoking may have biased our results. Second, even though our statin exposure information is based on filled prescriptions we cannot exclude some misclassification of statin use due to the absence of information about statin medication adherence (and reasons for discontinuation), and since we analyzed statins in a once-exposed, always exposed approach. Furthermore, we did not account for pre-existing musculoskeletal disorders that could potentially impact the likelihood of being prescribed statins, due to an assumed susceptibility to adverse drug reactions affecting the musculoskeletal system. Third, although our study had nationwide coverage during a period of almost 15 years, it is possible that a number of subgroup analyses, for example, analysis by statin type, were underpowered to detect the effects of statin use with smaller magnitudes. Fourth, confounding by indication cannot be ruled out since we did not have access to data on the actual indication of statin prescription. Fifth, patients who seek healthcare and get prescriptions for statins, might in general be more prone to follow treatments and recommendations. Patients who get a prescription for statins might also improve their lifestyle after recommendations from healthcare professionals. This potential healthy user bias would in that case be differential in our study and could potentially have an impact also on the risk of IBD outcomes. Sixth, although patients propensity scores matched by disease location (for CD), disease extent (for UC), disease duration, healthcare utilization, Charlson Comorbidity Index, as well as use of steroids, immunomodulators, and anti-TNF before index, we cannot rule out that nuances of IBD disease severity may have been imbalanced and may thus have impacted the risk of certain outcomes. Seventh, due to the lack of data on biochemical and endoscopic findings in the registers used in this study we were unable to include such data in our definition of disease flare. Finally, our study population was mainly of Caucasian origin and the setting of the study was a country that provides universal access to health care to all its residents, potentially limiting the generalizability to populations of other races in other healthcare settings.

In conclusion, we found that statins were associated with a reduced risk of IBD-related surgery, hospitalizations, and disease flares in patients with UC, and with a reduced risk of IBD-related surgery in patients with CD. Future studies are needed to confirm our findings and should focus on potential mechanisms that could explain the effect of statins on IBD outcomes and the potentially differential effect in UC and CD.

## Supplementary Data

Supplementary data is available at *Inflammatory Bowel Diseases* online.

izaf077_Supplementary_Material

## Data Availability

The data underlying this article cannot be shared publicly due to Swedish regulations. The findings presented in this manuscript, including related data, figures, and tables have not been previously published and the manuscript is not under consideration elsewhere.

## Abbreviations:

aHR: Adjusted hazard ratio
CI: Confidence interval
CD: Crohn’s disease
IBD-U: Inflammatory bowel disease unclassified
ICD: International classification of diseases
IQR: Interquartile range
NPR: National Patient Register
NNT: Number needed to treat
PPV: Positive predictive value
PDR: Prescribed Drug Register
LISA: Swedish Longitudinal, Integrated Database for Health Insurance and Labour Market Studies
TPR: Total Population Register
UC: Ulcerative colitis
