# Supplementary material for: Statin Use is Associated with a Less Severe Disease Course In Inflammatory Bowel Disease: A Nationwide Cohort Study 2006-2020
Source: Inflamm Bowel Dis. 2025 Apr 25;31(10):2787–97. doi: 10.1093/ibd/izaf077 (PMC12558581; doi:10.1093/ibd/izaf077)
Supplement: izaf077_Supplementary_Material [file izaf077_supplementary_material.docx]

# **Supplementary material**

This supplement contains additional tables and figures to the study by Khalili/Forss et al:

*"Statin use is associated with a less severe disease course in inflammatory bowel disease: A nationwide cohort study 2006-2020"*

**Table of contents**

**eMethods**

Data sources (pages 2)

Description of matching criteria (pages 2-3)

Calculation of numbers needed to treat (page 3)

**Table M1** Definitions of exposure, outcome, comorbidity, and prescription medication (pages 4-5)

**Supplementary tables and figures**

**Table S1a-b** Table of inclusion/exclusion of the cohorts (page 6)

**Table S2** Baseline characteristics of included patients with Crohn’s disease and ulcerative colitis exposed to statins before matching 2006-2020 (pages 7-9)

**Table S3a** Incidence rates and hazard ratios of inflammatory bowel disease related surgery in patients with ulcerative colitis with and without statin treatment 2006-2020 (page 10)

**Table S3b** Incidence rates and hazard ratios of inflammatory bowel disease related surgery in patients with Crohn’s disease with and without statin treatment 2006-2020 (page 11)

**Table S4a** Incidence rates and hazard ratios of inflammatory bowel disease related hospitalization in patients with ulcerative colitis with and without statin treatment 2006-2020 (page 12)

**Table S4b** Incidence rates and hazard ratios of inflammatory bowel disease related hospitalization in patients with Crohn’s disease with and without statin treatment 2006-2020 (page 13)

**Table S5a** Incidence rates and hazard ratios of disease flares (systemic steroid use, start of immunomodulator or start/switch of anti-TNF treatment) in patients with ulcerative colitis with and without statin treatment 2006-2020 (page 14)

**Table S5b** Incidence rates and hazard ratios of disease flares (systemic steroid use, start of immunomodulator or start/switch of anti-TNF treatment) in patients with Crohn’s disease with and without statin treatment 2006-2020 (page 15)

**References** (page 16)

**eMethods**

**Data sources**

The Swedish Total Population Register (TPR)^1^ was formed in 1968 and contains data on birth, death, migration on all Swedish residents. Exposure, outcome measures, and covariates were ascertained from the Swedish National Patient Register (NPR).^2^ The NPR includes prospectively recorded inpatient data including discharge diagnoses and procedure codes since 1964, with full nationwide coverage from 1987, and since 2001 includes also outpatient visits (except primary care). Clinical diagnoses in the NPR show positive predictive values (PPVs) between 85 and 95%, including surgical procedure codes.^2, 3^ Through the unique personal identity number^4^ assigned to all Swedish legal residents, we linked all identified patients with IBD to nationwide registers with data on demographics, migration, death, education level, medical diagnoses and prescribed medications. Education level was ascertained from the Longitudinal integrated database for health insurance and labour market studies (LISA).^5^ Data on prescription medications for four different categories of medications (antiarrhythmics, vasodilators, anti-thrombotic medication and statins, Table S3) was captured in the Prescribed Drug Register, a nationwide register with nearly full coverage^6^ of data for dispensed prescriptions since 1 July 2005. Data on inflammatory bowel disease medications (for the outcome of disease flare that used anti-tumor necrosis factor alpha inhibitors as part of the definition) was further strengthened by medication data in the Swedish Inflammatory Bowel Disease Register (SWIBREG).^7^

**Description of matching criteria**

Matching was performed by sampling 1:1 without replacement in 2 steps: 1) direct matching and 2) Propensity Score (PS)-matching.

After the first direct match step, index date in potential comparator patients with ulcerative colitis (UC) and Crohn’s disease (CD) were assigned using any clinic outpatient visit from the National Patient Register (any diagnosis) or prescription date from the Prescribed Drug Register (any prescription) ±2 weeks in relation to start date of statin treatment in the matched statin exposed patient with UC and CD. Definitions of exposure, outcome, comorbidity, and prescription medication are detailed in Table M1.

1. Direct matching

- Inflammatory bowel disease subtype (UC or CD)
- Sex (women, men)
- Age (<60y, ≥60y)
- Year of UC/CD diagnosis (2006-2010, 2011-2015, 2016-2019)
- Level of education (≤12y, >12y)

2. PS-matching (nearest neighbor algorithm) separately for UC and CD

- Sex (women, men)
- Age (continuous)
- Year of UC/CD diagnosis (2006-2010, 2011-2015, 2016-2019)
- Healthcare region (North, Central, Stockholm, West, South-east, South, Missing)
- Disease duration (continuous)
- Extent for UC (E1/E2, E3/EX)
- Disease location for CD (L2, L1/L3/LX)
- Number of outpatient healthcare visits 1 year before index date (continuous)
- Number of hospitalizations 1 year before index date (continuous)
- Number of prescriptions 1 year before index date (continuous)
- Country of birth (Nordic, non-Nordic)
- Level of education (≤9y, 10-12y, >12y, Missing)
- Comorbidities/Drugs (diabetes, hypertension, cardiovascular disease, metformin use, insulin use, aspirin use, ACE-inhibitor use)

Standardized difference

- Standardize difference between -0.10 to 0.10 is considered to indicate balance between stain users and statin non-users, hence, standardized difference ≤-0.10 or ≥0.10 indicate imbalance between the groups

**Calculation of numbers needed to treat**

Numbers needed to treat (NNT) was calculated for outcomes that reached statistically significant associations with statin use. NNT was estimated as the inverse of the incidence rate difference between non-users and statin users without further adjustment. Example for ulcerative colitis and surgical events:

Incidence rate non-statin users: 6.3 (95%CI 4.2-8.5) per 1000 person-years

Incidence rate statin users: 3.4 (95%CI 2.1-4.8) per 1000 person-years

Incidence rate difference: 6.3 - 3.4 = 2.9 per 1000 person-years

NNT: 1 / (2.9/1000) = 345 per 1 year of statin treatment

**eMethods**

**Table M1** Definitions of exposure, outcome, comorbidity, and prescription medication

|  | **ICD-8**  **(1969-1986)** | **ICD-9**  **(1987-1996)** | **ICD-10**  **(1997-)** | **Surgical Procedure code** | **ATC code** |
| --- | --- | --- | --- | --- | --- |
| **Primary disease** |  |  |  |  |  |
| **Crohn’s disease*** |  | 555^#^ | K50  Location:  Small intestine: K50.0  Large intestine: K50.1  Small and large: K50.8  Unspecified: K50.9  Behavior:  Without complications: K50.00, K50.10, K50.80, K50.90; Fistula:  K50.013, K50.113, K K50.813, K50.913; Stricture:  K50.012, K50.112, K K50.812, K50.912 | Includes bowel resections and related formation of stomas:  JFB00, JFB01  JFB20, JFB21  JFB30, JFB31  JFB33, JFB34  JFB40, JFB41  JFB43, JFB44  JFB46, JFB47  JFB50, JFB51  JFB60, JFB61  JFB63, JFB64  JFF10, JFF11  JFF23, JFF24  JFF26, JFF27  JFF30, JFF31  JFF96, JFF97  JFH00, JFH01  JFH10, JFH11  JFH20, JFH96 |  |
| **Ulcerative colitis*** |  | 556^#^ | K51  Extent:  Pan-colitis: K51.0  Proctitis: K51.2  Left-sided: K51.3, K51.5  Colitis unspecified: K51.4, K51.8, K51.9. | JFH00, JFH01  JFH10, JFH11  JFH20, JFH96  JGB50, JGB60 |  |
| **Comorbidity** |  |  |  |  |  |
| **Cardiovascular disease** | 391-399  410-449 | 391-459 | I05-09;  I20-I79  G45 |  | B01AA  B01AB  B01AC13-27  B01AD  B01AE  B01AF |
| **Metabolic Syndrome** |  |  |  |  |  |
| Diabetes (yes/no) | 250 | 250 | E10-E14  (E14 includes E14.0-E14.9) |  | A10A  A10AB-AE  A10BA-BX |
| Dyslipidemia (yes/no) | 272 | 272 | E78 |  | C10AA  C10BA  C10BX  C10AB  C10AC |
| Hypertension | 400-404 | 401*-405* | I10-I16 |  | C09A-CO9D  C09X  C01DA08-C01DA14  C03A-E |
| **Medication** |  |  |  |  |  |
| **IBD medications:**  Corticosteroids  Immuosuppressives  Anti-TNF |  |  |  |  | H02AB06, H02AB07, A07EA06  L01BA01, L01BB02, L04AX01  L04AB02, L04AB04 |
| **Other medications:**  Low-dose aspirin |  |  |  |  | B01AC06 |
| Statins |  |  |  |  | C10AA  C10BA  C10BX |
| Non-statin lipid-lowering drugs |  |  |  |  | C10AB (fibrates), C10AD (nicotinic acid), C10AX01-14 (other) |
| Glaucoma eye preparations |  |  |  |  | S01E |
| Metformin |  |  |  |  | A10BA02 |
| Pioglitazone |  |  |  |  | A10BG03 |
| ACE inhibitor or Angiotensin Receptor Blocker (ARB) |  |  |  |  | C09A-CO9D  C09X |

^*^The first diagnostic listing of CD or UC defined the subtype for the patient.
^#^Patients with a diagnostic listing of IBD before 2006 were excluded.

**Supplementary tables**

**Table S1a** Table of exclusion of patients with Crohn’s disease and ulcerative colitis from the source population 2006-2019

| **Exclusion** | **UC** | | | **CD** | | |
| --- | --- | --- | --- | --- | --- | --- |
|  | **N patients** | **N excluded** | **% excluded** | **N patients** | **N excluded** | **% excluded** |
| First ever diagnosis for UC or CD in the NPR in July 1, 2006 to December 31, 2019 | 41 138 |  |  | 23 949 |  |  |
|  |  |  |  |  |  |  |
| ***Exclusions I*** |  |  |  |  |  |  |
| No IBD drug use | 25 785 | 15 353 | 37% | 17 258 | 6 691 | 28% |
| Age <18 years at IBD diagnosis | 23 756 | 2 029 | 8% | 15 129 | 2 129 | 12% |
| Reused personal identity number | 23 737 | 19 | 0% | 15 110 | 19 | 0% |
| Diseased at index date | 23 269 | 468 | 2% | 14 875 | 235 | 2% |
| Emigration from IBD diagnosis to index date | 23 237 | 32 | 0% | 14 855 | 20 | 0% |
| Migration within 1 year before IBD diagnosis | 22 950 | 287 | 1% | 14 599 | 256 | 2% |
|  |  |  |  |  |  |  |
| ***Exclusions II*** |  |  |  |  |  |  |
| IBD-related surgery within 6 months before index date | 22 579 | 371 | 2% | 13 992 | 607 | 4% |
| Statin use within 12 months before IBD diagnosis | 20 024 | 2 555 | 11% | 12 694 | 1 298 | 9% |
| Statin use within first 6 months after IBD diagnosis | 19 788 | 236 | 1% | 12 582 | 112 | 1% |
|  |  |  |  |  |  |  |
| **Total** | **19 788** | **21 350** | **52** | **12 582** | **11 367** | **47** |

CD, Crohn’s disease; IBD, inflammatory bowel disease; NPR, national patient register; UC, ulcerative colitis

Index date: 6 months after IBD diagnosis.

**Table S1b** Table of inclusion of statin users Crohn’s disease and ulcerative colitis in the study population 2006-2019

| **Exclusion** | **UC** | | | **CD** | | |
| --- | --- | --- | --- | --- | --- | --- |
|  | **N patients** | **N excluded** | **% excluded** | **N patients** | **N excluded** | **% excluded** |
| Start of ≥30 concecutive cDDDs of statin use | 1 737 |  |  | 975 |  |  |
|  |  |  |  |  |  |  |
| ***Exclusions I*** |  |  |  |  |  |  |
| Diseased at index date | 1 736 | 1 | 0 | 975 | 0 | 0 |
| Migration within 1 year before index date | 1 735 | 1 | 0 | 974 | 1 | 0 |
|  |  |  |  |  |  |  |
| ***Exclusions II*** |  |  |  |  |  |  |
| IBD-related surgery within 6 months before index date | 1 733 | 2 | 0 | 962 | 12 | 1 |
|  |  |  |  |  |  |  |
| **Total** | **1 733** | **4** | **0** | **962** | **13** | **1** |

CD, Crohn’s disease; cDDD, cumulative daily d efined doses; IBD, inflammatory bowel disease; UC, ulcerative colitis

Index date: start of ≥30 consecutive cumulative daily defined doses of statin use

**Table S2** Baseline characteristics of included patients with Crohn’s disease and ulcerative colitis exposed to statins before matching

| **Characteristic** | **UC** | | **CD** | |
| --- | --- | --- | --- | --- |
|  | **Statin users**  **(N=1,733)** | **All UC patients**  **(N=19,788)** | **Statin users**  **(N=962)** | **All CD patients**  **(N=12,582)** |
| Sex, no. |  |  |  |  |
| Women | 782 (45.1%) | 9 729 (49.2%) | 486 (50.5%) | 6 777 (53.9%) |
| Men | 951 (54.9%) | 10 059 (50.8%) | 476 (49.5%) | 5 805 (46.1%) |
| Age at IBD diagnosis |  |  |  |  |
| Mean (SD) | 59.8 (13.0) | 44.3 (18.7) | 58.1 (13.3) | 42.3 (18.4) |
| Median (IQR) | 61.0 (51.9-69.0) | 40.6 (28.1-58.8) | 59.1 (49.4-67.3) | 38.7 (26.2-56.2) |
| *Categories, no.* |  |  |  |  |
| 18y - <40y | 132 (7.6%) | 9 687 (49.0%) | 92 (9.6%) | 6 559 (52.1%) |
| 40y - <50y | 246 (14.2%) | 2 902 (14.7%) | 164 (17.0%) | 1 859 (14.8%) |
| 50y - <60y | 433 (25.0%) | 2 539 (12.8%) | 241 (25.1%) | 1 569 (12.5%) |
| ≥60y | 922 (53.2%) | 4 660 (23.5%) | 465 (48.3%) | 2 595 (20.6%) |
| Age at index date* |  |  |  |  |
| Mean (SD) | 64.3 (12.5) | 44.8 (18.7) | 62.8 (12.8) | 42.8 (18.4) |
| Median (IQR) | 65.4 (56.1-73.1) | 41.1 (28.6-59.2) | 64.1 (54.2-72.1) | 39.2 (26.7-56.7) |
| *Categories, no.* |  |  |  |  |
| 18y - <40y | 62 (3.6%) | 9 510 (48.1%) | 48 (5.0%) | 6 464 (51.4%) |
| 40y - <50y | 169 (9.8%) | 2 968 (15.0%) | 107 (11.1%) | 1 868 (14.8%) |
| 50y - <60y | 359 (20.7%) | 2 527 (12.8%) | 222 (23.1%) | 1 578 (12.5%) |
| ≥60y | 1 143 (66.0%) | 4 783 (24.2%) | 585 (60.8%) | 2 672 (21.2%) |
| Healthcare region |  |  |  |  |
| North | 145 (8.4%) | 1 283 (6.5%) | 84 (8.7%) | 739 (5.9%) |
| Central | 439 (25.3%) | 5 275 (26.7%) | 224 (23.3%) | 3 338 (26.5%) |
| Stockholm | 247 (14.3%) | 2 872 (14.5%) | 194 (20.2%) | 2 169 (17.2%) |
| West | 378 (21.8%) | 3 325 (16.8%) | 129 (13.4%) | 1 452 (11.5%) |
| South-east | 169 (9.8%) | 1 598 (8.1%) | 100 (10.4%) | 1 049 (8.3%) |
| South | 334 (19.3%) | 4 830 (24.4%) | 223 (23.2%) | 3 385 (26.9%) |
| Missing | 21 (1.2%) | 605 (3.1%) | 8 (0.8%) | 450 (3.6%) |
| Country of birth, no. |  |  |  |  |
| Nordic country | 1 587 (91.6%) | 17 612 (89.0%) | 842 (87.5%) | 10 778 (85.7%) |
| Other European country | 59 (3.4%) | 902 (4.6%) | 46 (4.8%) | 703 (5.6%) |
| Other non-European country | 87 (5.0%) | 1 273 (6.4%) | 74 (7.7%) | 1 100 (8.7%) |
| Missing | 0 | 1 (0.0%) | 0 | 1 (0.0%) |
| Level of education, no. |  |  |  |  |
| ≤9 y | 485 (28.0%) | 3 779 (19.1%) | 277 (28.8%) | 2 625 (20.9%) |
| 10-12 y | 861 (49.7%) | 9 529 (48.2%) | 451 (46.9%) | 6 089 (48.4%) |
| >12 y | 377 (21.8%) | 6 324 (32.0%) | 226 (23.5%) | 3 749 (29.8%) |
| Missing | 10 (0.6%) | 156 (0.8%) | 8 (0.8%) | 119 (0.9%) |
| Year of IBD diagnosis, no. |  |  |  |  |
| 2006-2010 | 749 (43.2%) | 5 499 (27.8%) | 451 (46.9%) | 3 348 (26.6%) |
| 2011-2015 | 711 (41.0%) | 7 471 (37.8%) | 367 (38.1%) | 4 768 (37.9%) |
| 2016-2019 | 273 (15.8%) | 6 818 (34.5%) | 144 (15.0%) | 4 466 (35.5%) |
| Year of index date*, no. |  |  |  |  |
| 2006-2010 | 148 (8.5%) | 4 825 (24.4%) | 80 (8.3%) | 2 965 (23.6%) |
| 2011-2015 | 535 (30.9%) | 7 388 (37.3%) | 313 (32.5%) | 4 688 (37.3%) |
| 2016-2020 | 1 050 (60.6%) | 7 575 (38.3%) | 569 (59.1%) | 4 929 (39.2%) |
| Montreal classification CD |  |  |  |  |
| L2 |  |  | 156 (16.2%) | 2 448 (19.5%) |
| L1/L3/LX |  |  | 806 (83.8%) | 10 134 (80.5%) |
| B1 |  |  | 814 (84.6%) | 11 158 (88.7%) |
| B2/B3 |  |  | 148 (15.4%) | 1 424 (11.3%) |
| Perianal |  |  | 62 (6.4%) | 962 (7.6%) |
| Montreal classification UC |  |  |  |  |
| E1/E2 | 606 (35.0%) | 7 553 (38.2%) |  |  |
| E3 | 368 (21.2%) | 5 230 (26.4%) |  |  |
| EX | 756 (43.6%) | 6 990 (35.3%) |  |  |
| Missing | 3 (0.2%) | 15 (0.1%) |  |  |
| Extraintestinal manifestations (EIM) |  |  |  |  |
| Primary sclerosing cholangitis | 23 (1.3%) | 323 (1.6%) | 13 (1.4%) | 104 (0.8%) |
| Other (EIM) | 316 (18.2%) | 2 218 (11.2%) | 240 (24.9%) | 1 750 (13.9%) |
| Comorbidities ever before index date*, no. |  |  |  |  |
| Diabetes | 496 (28.6%) | 913 (4.6%) | 266 (27.7%) | 494 (3.9%) |
| Hypertension | 1 249 (72.1%) | 3 897 (19.7%) | 691 (71.8%) | 2 432 (19.3%) |
| Dyslipidemia | 1 733 (100.0%) | 1 380 (7.0%) | 962 (100.0%) | 1 144 (9.1%) |
| Cardiovascular disease | 991 (57.2%) | 3 533 (17.9%) | 552 (57.4%) | 2 187 (17.4%) |
| Medications ever before index date*, no. |  |  |  |  |
| Metformin use | 360 (20.8%) | 473 (2.4%) | 183 (19.0%) | 271 (2.2%) |
| Insulin use | 197 (11.4%) | 477 (2.4%) | 110 (11.4%) | 225 (1.8%) |
| Aspirin use | 728 (42.0%) | 1 325 (6.7%) | 420 (43.7%) | 799 (6.4%) |
| ACE inhibitor use | 1 037 (59.8%) | 2 587 (13.1%) | 554 (57.6%) | 1 538 (12.2%) |
| Date of IBD diagnosis, no. |  |  |  |  |
| UC or CD diagnosis from NPR | 739 (42.6%) | 7 401 (37.4%) | 410 (42.6%) | 4 951 (39.3%) |
| IBD drug from PDR | 994 (57.4%) | 12 387 (62.6%) | 552 (57.4%) | 7 631 (60.7%) |
| Systemic steroid use | 963 (55.6%) | 11 955 (60.4%) | 510 (53.0%) | 6 936 (55.1%) |
| Immunosuppressive medication | 23 (1.3%) | 218 (1.1%) | 36 (3.7%) | 481 (3.8%) |
| Anti-TNF | 8 (0.5%) | 214 (1.1%) | 6 (0.6%) | 214 (1.7%) |
| Outcomes ≤6 months of index date* |  |  |  |  |
| IBD-related hospitalization | 25 (1.4%) | 2 141 (10.8%) | 18 (1.9%) | 1 329 (10.6%) |
| Systemic steroid use | 442 (25.5%) | 15 782 (79.8%) | 291 (30.2%) | 10 021 (79.6%) |
| Immunosuppressive medication | 201 (11.6%) | 3 538 (17.9%) | 144 (15.0%) | 4 317 (34.3%) |
| Started anti-TNF | 17 (1.0%) | 1 175 (5.9%) | 18 (1.9%) | 1 405 (11.2%) |
| Healthcare utilization ≤1 year before index date* |  |  |  |  |
| *Number of outpatient care visits* |  |  |  |  |
| Mean (SD) | 4.0 (4.4) | 4.5 (4.2) | 4.9 (7.9) | 5.5 (4.8) |
| Median (IQR) | 3 (1-5) | 3 (2-6) | 3 (1-6) | 4 (3-7) |
| *Number of hospitalizations* |  |  |  |  |
| Mean (SD) | 0.8 (1.4) | 0.8 (1.4) | 1.1 (1.8) | 1.0 (1.6) |
| Median (IQR) | 0 (0-1) | 0 (0-1) | 0 (0-1) | 0 (0-1) |
| *Number of prescription medications* |  |  |  |  |
| Mean (SD) | 35.5 (42.7) | 23.6 (29.6) | 36.2 (33.6) | 23.7 (32.5) |
| Median (IQR) | 26 (16-40) | 17 (10-27) | 28 (16-44) | 15 (9-26) |
| Charlson Comorbidity Index |  |  |  |  |
| Mean (SD) | 1.5 (1.8) | 0.6 (1.2) | 1.5 (1.7) | 0.5 (1.1) |
| Median (IQR) | 1 (0-2) | 0 (0-1) | 1 (0-2) | 0 (0-1) |
| Range, min-max | 0-13 | 0-13 | 0-11 | 0-11 |
| *Categories, no.* |  |  |  |  |
| 0 | 633 (36.5%) | 14 410 (72.8%) | 319 (33.2%) | 8 869 (70.5%) |
| 1 | 411 (23.7%) | 2 727 (13.8%) | 251 (26.1%) | 2 093 (16.6%) |
| 2 | 319 (18.4%) | 1 391 (7.0%) | 171 (17.8%) | 900 (7.2%) |
| ≥3 | 370 (21.4%) | 1 260 (6.4%) | 221 (23.0%) | 720 (5.7%) |
| Type of statin drug at treatment start, no. |  |  |  |  |
| Simvastatin | 567 (32.7%) | - | 329 (34.2%) | - |
| Atorvastatin | 1 080 (62.3%) | - | 589 (61.2%) | - |
| Rosuvastatin | 79 (4.6%) | - | 37 (3.8%) | - |
| Pravastatin | 7 (0.4%) | - | 7 (0.7%) | - |
| Disease duration at index date* |  |  |  |  |
| Mean (SD) | 4.5 (3.1) | 0.5 (0.0) | 4.7 (3.3) | 0.5 (0.0) |
| Median (IQR) | 3.8 (1.8-6.4) | 0.5 (0.5-0.5) | 4.0 (1.8-7.0) | 0.5 (0.5-0.5) |
| *Categories, no.* |  |  |  |  |
| <1y | 168 (9.7%) | 19 788 (100%) | 108 (11.2%) | 12 582 (100%) |
| 1-<5y | 906 (52.3%) | 0 | 481 (50.0%) | 0 |
| 5-<10y | 544 (31.4%) | 0 | 296 (30.8%) | 0 |
| ≥10y | 115 (6.6%) | 0 | 77 (8.0%) | 0 |
| Follow-up from index date* to death, migration, start of ≥30 consecutive cumulative DDDs or end of data, years |  |  |  |  |
| Mean (SD) | 4.1 (3.2) | 5.9 (3.8) | 4.2 (3.2) | 5.9 (3.7) |
| Median (IQR) | 3.4 (1.4-6.1) | 5.4 (2.6-8.9) | 3.4 (1.4-6.3) | 5.4 (2.6-8.8) |
| *Categories, no.* |  |  |  |  |
| <1y | 302 (17.4%) | 1 461 (7.4%) | 174 (18.1%) | 932 (7.4%) |
| 1-<5y | 855 (49.3%) | 7 747 (39.1%) | 443 (46.0%) | 4 938 (39.2%) |
| 5-<10y | 463 (26.7%) | 6 913 (34.9%) | 283 (29.4%) | 4 441 (35.3%) |
| ≥10y | 113 (6.5%) | 3 667 (18.5%) | 62 (6.4%) | 2 271 (18.0%) |
| *Reason for end of follow-up, no. (%)* |  |  |  |  |
| Death | 198 (11.4%) | 1 305 (6.6%) | 118 (12.3%) | 729 (5.8%) |
| Emigration | 11 (0.6%) | 258 (1.3%) | 5 (0.5%) | 196 (1.6%) |
| Start of statin ≥30 consecutive cDDDs | - | 1 734 (8.8%) | - | 973 (7.7%) |
| End of data (Dec 31, 2020) | 1 524 (87.9%) | 16 491 (83.3%) | 839 (87.2%) | 10 684 (84.9%) |
|  |  |  |  |  |

ACE inhibitor, angiotensin-converting-enzyme inhibitor; anti-TNF; anti-tumor necrosis factor alpha; CD, Crohn’s disease; cDDD, cumulative defined daily dose; DDD, defined daily dose; IBD, inflammatory bowel disease, IQR, interquartile range; NPR; national patient register; PDR; prescribed drug register; SD, standard deviation; UC, ulcerative colitis; y, years

*Index date: >6 months after IBD diagnosis and start of ≥30 consecutive cumulative DDDs in statin users with UC and CD.

**Table S3a** Incidence rates and hazard ratios of inflammatory bowel disease related surgery in patients with ulcerative colitis with and without statin treatment 2006-2020

| **Outcome** | **N** | | | **N events** | | | **Incidence rate (95% CI) per 1000 PY** | | **HR***  **(95%CI)** | **aHR****  **(95%CI)** |
| --- | --- | --- | --- | --- | --- | --- | --- | --- | --- | --- |
|  | **Statin users** | **Non-statin users** | **Statin users** | | **Non-statin users** | **Statin users** | | **Non-statin users** |  |  |
| **Overall** | 1 733 (100%) | 1 733 (100%) | 24 (1.4%) | | 34 (2.0%) | 3.4 (2.1-4.8) | | 6.3 (4.2-8.5) | 0.56 (0.33-0.95) | 0.55 (0.31-0.97) |
| Follow-up time |  |  |  | |  |  | |  |  |  |
| <1y | 1 733 (100%) | 1 733 (100%) | 7 (0.4%) | | 13 (0.8%) | 4.4 (1.2-7.7) | | 8.6 (3.9-13.3) | 0.52 (0.21-1.29) | 0.54 (0.21-1.35) |
| 1-<5y | 1 424 (82.2%) | 1 295 (74.7%) | 12 (0.8%) | | 15 (1.2%) | 3.2 (1.4-5.0) | | 5.0 (2.5-7.5) | 0.64 (0.30-1.39) | 0.57 (0.24-1.36) |
| ≥5y | 562 (32.4%) | 377 (21.8%) | 5 (0.9%) | | 6 (1.6%) | 1.1 (0.1-2.1) | | 2.2 (0.4-3.9) | 0.46 (0.14-1.51) | 0.50 (0.13-2.00) |
| Sex |  |  |  | |  |  | |  |  |  |
| Women | 782 (45.1%) | 782 (45.1%) | 6 (0.8%) | | 16 (2.0%) | 1.9 (0.4-3.5) | | 6.5 (3.3-9.7) | 0.28 (0.11-0.70) | 0.25 (0.08-0.75) |
| Men | 951 (54.9%) | 951 (54.9%) | 18 (1.9%) | | 18 (1.9%) | 4.6 (2.5-6.8) | | 6.1 (3.3-9.0) | 0.83 (0.43-1.62) | 0.82 (0.41-1.67) |
| Age |  |  |  | |  |  | |  |  |  |
| 18-<60y | 590 (34.0%) | 637 (36.8%) | 12 (2.0%) | | 15 (2.4%) | 4.5 (2.0-7.1) | | 6.5 (3.2-9.8) | 0.70 (0.33-1.49) | 0.73 (0.29-1.81) |
| ≥60y | 1 143 (66.0%) | 1 096 (63.2%) | 12 (1.0%) | | 19 (1.7%) | 2.8 (1.2-4.4) | | 6.2 (3.4-9.0) | 0.48 (0.23-1.02) | 0.56 (0.25-1.27) |
| IBD diagnosis |  |  |  | |  |  | |  |  |  |
| 2006-2010 | 749 (43.2%) | 749 (43.2%) | 11 (1.5%) | | 18 (2.4%) | 2.6 (1.1-4.2) | | 6.0 (3.3-8.8) | 0.44 (0.21-0.93) | 0.33 (0.13-0.84) |
| 2011-2015 | 711 (41.0%) | 711 (41.0%) | 12 (1.7%) | | 12 (1.7%) | 5.0 (2.2-7.9) | | 5.8 (2.5-9.2) | 0.88 (0.39-1.98) | 0.92 (0.40-2.08) |
| 2016-2019 | 273 (15.8%) | 273 (15.8%) | 1 (0.4%) | | 4 (1.5%) | 2.6 (0.0-7.7) | | 11.5 (0.2-22.8) | 0.24 (0.03-2.14) | 0.33 (0.03-3.20) |
| Start year of follow-up |  |  |  | |  |  | |  |  |  |
| 2006-2010 | 148 (8.5%) | 146 (8.4%) | 6 (4.1%) | | 8 (5.5%) | 4.3 (0.9-7.8) | | 9.4 (2.9-16.0) | 0.48 (0.16-1.42) | 0.25 (0.05-1.18) |
| 2011-2015 | 535 (30.9%) | 537 (31.0%) | 14 (2.6%) | | 14 (2.6%) | 4.2 (2.0-6.5) | | 5.6 (2.6-8.5) | 0.83 (0.39-1.76) | 0.93 (0.44-1.98) |
| 2016-2020 | 1 050 (60.6%) | 1 050 (60.6%) | 4 (0.4%) | | 12 (1.1%) | 1.8 (0.0-3.5) | | 6.0 (2.6-9.4) | 0.29 (0.09-0.91) | 0.27 (0.08-0.98) |
| Level of education |  |  |  | |  |  | |  |  |  |
| ≤12y | 1 346 (77.7%) | 1 349 (77.8%) | 20 (1.5%) | | 28 (2.1%) | 3.6 (2.0-5.2) | | 6.6 (4.2-9.1) | 0.57 (0.32-1.02) | 0.54 (0.28-1.03) |
| >12y | 377 (21.8%) | 377 (21.8%) | 4 (1.1%) | | 6 (1.6%) | 3.0 (0.1-5.9) | | 5.3 (1.1-9.5) | 0.55 (0.16-1.93) | 0.67 (0.19-2.36) |
| Type of statin drug at treatment start |  |  |  | |  |  | |  |  |  |
| Simvastatin | 567 (32.7%) | 567 (32.7%) | 12 (2.1%) | | 20 (3.5%) | 3.3 (1.4-5.2) | | 7.8 (4.4-11.1) | 0.45 (0.22-0.93) | 0.35 (0.15-0.83) |
| Atorvastatin | 1 080 (62.3%) | 1 080 (62.3%) | 11 (1.0%) | | 13 (1.2%) | 3.5 (1.4-5.5) | | 4.9 (2.2-7.5) | 0.73 (0.32-1.63) | 0.83 (0.36-1.93) |
| Other statins | 86 (5.0%) | 86 (5.0%) | 1 (1.2%) | | 1 (1.2%) | 6.6 (0.0-19.7) | | 7.4 (0.0-22.0) | 0.78 (0.04-13.61) | 1.00 (0.06-15.99) |

aHR, adjusted hazard ratio; CI, confidence interval; HR, hazard ratio; PY, person-years; y, years

*Propensity score matched for sex, age, level of education, year of diagnosis, disease duration, location and extent, healthcare utilization, and comorbidities.

**Conditioned on matching set

**Table S3b** Incidence rates and hazard ratios of inflammatory bowel disease related surgery in patients with Crohn’s disease with and without statin treatment 2006-2020

| **Outcome** | **N** | | | **N events** | | | **Incidence rate (95% CI) per 1000 PY** | | **HR***  **(95%CI)** | **aHR****  **(95%CI)** |
| --- | --- | --- | --- | --- | --- | --- | --- | --- | --- | --- |
|  | **Statin users** | **Non-statin users** | **Statin users** | | **Non-statin users** | **Statin users** | | **Non-statin users** |  |  |
| **Overall** | 962 (100%) | 962 (100%) | 36 (3.7%) | | 48 (5.0%) | 9.2 (6.2-12.2) | | 15.4 (11.0-19.7) | 0.59 (0.39-0.90) | 0.54 (0.33-0.88) |
| Follow-up time |  |  |  | |  |  | |  |  |  |
| <1y | 962 (100%) | 962 (100%) | 5 (0.5%) | | 15 (1.6%) | 5.7 (0.7-10.7) | | 17.8 (8.8-26.8) | 0.32 (0.12-0.90) | 0.33 (0.12-0.92) |
| 1-<5y | 786 (81.7%) | 733 (76.2%) | 16 (2.0%) | | 26 (3.5%) | 7.5 (3.8-11.2) | | 15.0 (9.2-20.8) | 0.50 (0.28-0.92) | 0.48 (0.24-0.96) |
| ≥5y | 335 (34.8%) | 231 (24.0%) | 15 (4.5%) | | 7 (3.0%) | 5.8 (2.9-8.8) | | 4.1 (1.1-7.2) | 1.30 (0.52-3.25) | 1.33 (0.46-3.84) |
| Sex |  |  |  | |  |  | |  |  |  |
| Women | 486 (50.5%) | 486 (50.5%) | 17 (3.5%) | | 22 (4.5%) | 8.8 (4.6-12.9) | | 13.5 (7.9-19.2) | 0.64 (0.34-1.20) | 0.70 (0.35-1.39) |
| Men | 476 (49.5%) | 476 (49.5%) | 19 (4.0%) | | 26 (5.5%) | 9.7 (5.3-14.0) | | 17.4 (10.7-24.1) | 0.54 (0.31-0.95) | 0.42 (0.21-0.86) |
| Age |  |  |  | |  |  | |  |  |  |
| 18-<60y | 377 (39.2%) | 384 (39.9%) | 14 (3.7%) | | 17 (4.4%) | 8.5 (4.1-13.0) | | 12.3 (6.5-18.2) | 0.69 (0.35-1.39) | 0.64 (0.28-1.49) |
| ≥60y | 585 (60.8%) | 578 (60.1%) | 22 (3.8%) | | 31 (5.4%) | 9.7 (5.7-13.8) | | 17.8 (11.5-24.1) | 0.53 (0.31-0.89) | 0.44 (0.23-0.88) |
| IBD diagnosis |  |  |  | |  |  | |  |  |  |
| 2006-2010 | 451 (46.9%) | 451 (46.9%) | 26 (5.8%) | | 19 (4.2%) | 10.7 (6.6-14.8) | | 10.0 (5.5-14.4) | 1.01 (0.55-1.84) | 0.95 (0.50-1.81) |
| 2011-2015 | 367 (38.1%) | 367 (38.1%) | 8 (2.2%) | | 23 (6.3%) | 6.4 (2.0-10.8) | | 22.6 (13.3-31.8) | 0.29 (0.14-0.60) | 0.24 (0.09-0.63) |
| 2016-2019 | 144 (15.0%) | 144 (15.0%) | 2 (1.4%) | | 6 (4.2%) | 9.2 (0.0-22.0) | | 31.0 (6.2-55.7) | 0.31 (0.06-1.58) | 0.33 (0.07-1.65) |
| Start year of follow-up |  |  |  | |  |  | |  |  |  |
| 2006-2010 | 80 (8.3%) | 80 (8.3%) | 11 (13.8%) | | 6 (7.5%) | 14.8 (6.1-23.6) | | 10.8 (2.2-19.4) | 1.28 (0.46-3.59) | 1.33 (0.46-3.84) |
| 2011-2015 | 313 (32.5%) | 313 (32.5%) | 17 (5.4%) | | 23 (7.3%) | 8.6 (4.5-12.7) | | 15.3 (9.0-21.5) | 0.56 (0.31-1.03) | 0.45 (0.22-0.96) |
| 2016-2020 | 569 (59.1%) | 569 (59.1%) | 8 (1.4%) | | 19 (3.3%) | 6.7 (2.1-11.4) | | 18.0 (9.9-26.0) | 0.38 (0.17-0.84) | 0.39 (0.16-0.93) |
| Level of education |  |  |  | |  |  | |  |  |  |
| ≤12y | 728 (75.7%) | 727 (75.6%) | 34 (4.7%) | | 35 (4.8%) | 11.5 (7.6-15.4) | | 15.0 (10.0-20.0) | 0.75 (0.47-1.19) | 0.70 (0.41-1.19) |
| >12y | 226 (23.5%) | 226 (23.5%) | 2 (0.9%) | | 13 (5.8%) | 2.2 (0.0-5.2) | | 17.2 (7.8-26.5) | 0.13 (0.03-0.52) | 0.17 (0.04-0.74) |
| Type of statin drug at treatment start |  |  |  | |  |  | |  |  |  |
| Simvastatin | 329 (34.2%) | 329 (34.2%) | 25 (7.6%) | | 22 (6.7%) | 12.2 (7.4-17.0) | | 14.2 (8.2-20.1) | 0.85 (0.49-1.50) | 0.86 (0.46-1.61) |
| Atorvastatin | 589 (61.2%) | 589 (61.2%) | 11 (1.9%) | | 24 (4.1%) | 6.4 (2.6-10.2) | | 16.7 (10.0-23.3) | 0.37 (0.19-0.74) | 0.30 (0.13-0.71) |
| Other statins | 44 (4.6%) | 44 (4.6%) | 0 | | 0 | 0 | | 0 | - | - |
| Other statins | 86 (5.0%) | 86 (5.0%) | 5 (5.8%) | | 2 (2.3%) | 35.7 (4.4-67.1) | | 15.6 (0.0-37.3) | 2.52 (0.48-13.34) | 2.50 (0.49-12.89) |

aHR, adjusted hazard ratio; CI, confidence interval; HR, hazard ratio; PY, person-years; y, years

*Propensity score matched for sex, age, level of education, year of diagnosis, disease duration, location and extent, healthcare utilization, and comorbidities

**Conditioned on matching set

**Table S4a** Incidence rates and hazard ratios of inflammatory bowel disease related hospitalization in patients with ulcerative colitis with and without statin treatment 2006-2020

| **Outcome** | **N** | | | **N events** | | | **Incidence rate (95% CI) per 1000 PY** | | **HR***  **(95%CI)** | **aHR****  **(95%CI)** |
| --- | --- | --- | --- | --- | --- | --- | --- | --- | --- | --- |
|  | **Statin users** | **Non-statin users** | **Statin users** | | **Non-statin users** | **Statin users** | | **Non-statin users** |  |  |
| **Overall** | 1 733 (100%) | 1 733 (100%) | 113 (6.5%) | | 121 (7.0%) | 17.0 (13.9-20.2) | | 23.9 (19.6-28.1) | 0.77 (0.59-0.99) | 0.68 (0.51-0.91) |
| Follow-up time |  |  |  | |  |  | |  |  |  |
| <1y | 1 733 (100%) | 1 733 (100%) | 39 (2.3%) | | 54 (3.1%) | 25.0 (17.2-32.9) | | 36.5 (26.8-46.3) | 0.69 (0.46-1.04) | 0.65 (0.42-0.99) |
| 1-<5y | 1 396 (80.6%) | 1 258 (72.6%) | 51 (3.7%) | | 61 (4.8%) | 14.0 (10.1-17.8) | | 21.6 (16.1-27.0) | 0.67 (0.46-0.97) | 0.64 (0.42-0.99) |
| ≥5y | 529 (30.5%) | 344 (19.8%) | 23 (4.3%) | | 6 (1.7%) | 5.6 (3.3-7.9) | | 2.4 (0.5-4.3) | 2.18 (0.90-5.29) | 1.40 (0.44-4.41) |
| Sex |  |  |  | |  |  | |  |  |  |
| Women | 782 (45.1%) | 782 (45.1%) | 30 (3.8%) | | 51 (6.5%) | 10.0 (6.4-13.6) | | 22.1 (16.0-28.2) | 0.48 (0.31-0.75) | 0.35 (0.20-0.62) |
| Men | 951 (54.9%) | 951 (54.9%) | 83 (8.7%) | | 70 (7.4%) | 22.8 (17.9-27.7) | | 25.3 (19.4-31.3) | 0.98 (0.71-1.35) | 0.92 (0.65-1.31) |
| Age |  |  |  | |  |  | |  |  |  |
| 18-<60y | 590 (34.0%) | 637 (36.8%) | 38 (6.4%) | | 55 (8.6%) | 15.1 (10.3-20.0) | | 25.6 (18.8-32.4) | 0.64 (0.42-0.96) | 0.74 (0.45-1.23) |
| ≥60y | 1 143 (66.0%) | 1 096 (63.2%) | 75 (6.6%) | | 66 (6.0%) | 18.2 (14.1-22.3) | | 22.6 (17.1-28.0) | 0.87 (0.62-1.21) | 0.71 (0.47-1.05) |
| IBD diagnosis |  |  |  | |  |  | |  |  |  |
| 2006-2010 | 749 (43.2%) | 749 (43.2%) | 72 (9.6%) | | 62 (8.3%) | 18.3 (14.1-22.5) | | 22.4 (16.8-27.9) | 0.90 (0.64-1.26) | 0.74 (0.50-1.10) |
| 2011-2015 | 711 (41.0%) | 711 (41.0%) | 29 (4.1%) | | 48 (6.8%) | 12.4 (7.9-17.0) | | 24.4 (17.5-31.4) | 0.53 (0.33-0.84) | 0.50 (0.30-0.83) |
| 2016-2019 | 273 (15.8%) | 273 (15.8%) | 12 (4.4%) | | 11 (4.0%) | 32.3 (14.0-50.6) | | 32.5 (13.3-51.7) | 1.01 (0.44-2.34) | 1.10 (0.47-2.59) |
| Start year of follow-up |  |  |  | |  |  | |  |  |  |
| 2006-2010 | 148 (8.5%) | 146 (8.4%) | 21 (14.2%) | | 21 (14.4%) | 16.2 (9.3-23.1) | | 27.7 (15.9-39.6) | 0.66 (0.36-1.21) | 0.45 (0.20-0.99) |
| 2011-2015 | 535 (30.9%) | 537 (31.0%) | 58 (10.8%) | | 51 (9.5%) | 18.6 (13.8-23.4) | | 21.5 (15.6-27.4) | 0.95 (0.65-1.38) | 0.83 (0.54-1.27) |
| 2016-2020 | 1 050 (60.6%) | 1 050 (60.6%) | 34 (3.2%) | | 49 (4.7%) | 15.3 (10.1-20.4) | | 25.2 (18.2-32.3) | 0.62 (0.40-0.97) | 0.63 (0.40-1.00) |
| Level of education |  |  |  | |  |  | |  |  |  |
| ≤12y | 1 346 (77.7%) | 1 349 (77.8%) | 99 (7.4%) | | 97 (7.2%) | 18.8 (15.1-22.5) | | 24.4 (19.5-29.2) | 0.83 (0.62-1.10) | 0.73 (0.53-1.00) |
| >12y | 377 (21.8%) | 377 (21.8%) | 14 (3.7%) | | 23 (6.1%) | 10.6 (5.0-16.1) | | 21.3 (12.6-30.1) | 0.55 (0.28-1.06) | 0.55 (0.27-1.10) |
| Type of statin drug at treatment start |  |  |  | |  |  | |  |  |  |
| Simvastatin | 567 (32.7%) | 567 (32.7%) | 57 (10.1%) | | 54 (9.5%) | 16.8 (12.4-21.1) | | 22.5 (16.5-28.5) | 0.83 (0.58-1.20) | 0.71 (0.46-1.10) |
| Atorvastatin | 1 080 (62.3%) | 1 080 (62.3%) | 51 (4.7%) | | 65 (6.0%) | 16.5 (12.0-21.0) | | 25.5 (19.3-31.7) | 0.66 (0.46-0.96) | 0.60 (0.40-0.90) |
| Other statins | 86 (5.0%) | 86 (5.0%) | 5 (5.8%) | | 2 (2.3%) | 35.7 (4.4-67.1) | | 15.6 (0.0-37.3) | 2.52 (0.48-13.34) | 2.50 (0.49-12.89) |

aHR, adjusted hazard ratio; CI, confidence interval; HR, hazard ratio; PY, person-years; y, years

*Propensity score matched for sex, age, level of education, year of diagnosis, disease duration, location and extent, healthcare utilization, and comorbidities

**Conditioned on matching set

**Table S4b** Incidence rates and hazard ratios of inflammatory bowel disease related hospitalization in patients with Crohn’s disease with and without statin treatment 2006-2020

| **Outcome** | **N** | | | **N events** | | | **Incidence rate (95% CI) per 1000 PY** | | **HR***  **(95%CI)** | **aHR****  **(95%CI)** |
| --- | --- | --- | --- | --- | --- | --- | --- | --- | --- | --- |
|  | **Statin users** | **Non-statin users** | **Statin users** | | **Non-statin users** | **Statin users** | | **Non-statin users** |  |  |
| **Overall** | 962 (100%) | 962 (100%) | 76 (7.9%) | | 84 (8.7%) | 20.3 (15.8-24.9) | | 28.2 (22.2-34.2) | 0.78 (0.57-1.06) | 0.78 (0.56-1.09) |
| Follow-up time, years |  |  |  | |  |  | |  |  |  |
| <1y | 962 (100%) | 962 (100%) | 30 (3.1%) | | 44 (4.6%) | 34.8 (22.3-47.2) | | 53.2 (37.5-68.9) | 0.66 (0.41-1.06) | 0.70 (0.44-1.11) |
| 1-<5y | 766 (79.6%) | 706 (73.4%) | 37 (4.8%) | | 33 (4.7%) | 18.3 (12.4-24.2) | | 20.1 (13.2-26.9) | 0.92 (0.58-1.47) | 0.90 (0.53-1.52) |
| ≥5y | 309 (32.1%) | 217 (22.6%) | 9 (2.9%) | | 7 (3.2%) | 3.7 (1.3-6.2) | | 4.4 (1.1-7.7) | 0.77 (0.28-2.12) | 0.80 (0.21-2.98) |
| Sex |  |  |  | |  |  | |  |  |  |
| Women | 486 (50.5%) | 486 (50.5%) | 41 (8.4%) | | 38 (7.8%) | 22.3 (15.4-29.1) | | 24.4 (16.6-32.1) | 0.96 (0.62-1.49) | 1.00 (0.62-1.61) |
| Men | 476 (49.5%) | 476 (49.5%) | 35 (7.4%) | | 46 (9.7%) | 18.4 (12.3-24.5) | | 32.4 (23.0-41.8) | 0.62 (0.40-0.96) | 0.60 (0.37-0.98) |
| Age |  |  |  | |  |  | |  |  |  |
| 18-<60y | 377 (39.2%) | 384 (39.9%) | 33 (8.8%) | | 43 (11.2%) | 21.2 (13.9-28.4) | | 33.4 (23.4-43.4) | 0.69 (0.44-1.09) | 0.79 (0.45-1.37) |
| ≥60y | 585 (60.8%) | 578 (60.1%) | 43 (7.4%) | | 41 (7.1%) | 19.7 (13.8-25.6) | | 24.3 (16.8-31.7) | 0.84 (0.56-1.29) | 0.77 (0.45-1.32) |
| IBD diagnosis |  |  |  | |  |  | |  |  |  |
| 2006-2010 | 451 (46.9%) | 451 (46.9%) | 39 (8.6%) | | 46 (10.2%) | 16.8 (11.5-22.0) | | 25.6 (18.2-33.0) | 0.71 (0.46-1.10) | 0.72 (0.45-1.14) |
| 2011-2015 | 367 (38.1%) | 367 (38.1%) | 29 (7.9%) | | 32 (8.7%) | 24.1 (15.3-32.9) | | 32.4 (21.2-43.7) | 0.79 (0.48-1.28) | 0.79 (0.45-1.37) |
| 2016-2019 | 144 (15.0%) | 144 (15.0%) | 8 (5.6%) | | 6 (4.2%) | 37.7 (11.6-63.7) | | 30.7 (6.1-55.2) | 1.26 (0.46-3.46) | 1.17 (0.39-3.47) |
| Start year of follow-up |  |  |  | |  |  | |  |  |  |
| 2006-2010 | 80 (8.3%) | 80 (8.3%) | 10 (12.5%) | | 13 (16.3%) | 13.6 (5.2-22.1) | | 25.7 (11.7-39.6) | 0.57 (0.25-1.31) | 0.58 (0.23-1.48) |
| 2011-2015 | 313 (32.5%) | 313 (32.5%) | 38 (12.1%) | | 37 (11.8%) | 20.6 (14.1-27.2) | | 25.7 (17.5-34.0) | 0.87 (0.55-1.36) | 0.85 (0.51-1.40) |
| 2016-2020 | 569 (59.1%) | 569 (59.1%) | 28 (4.9%) | | 34 (6.0%) | 24.1 (15.2-33.0) | | 32.9 (21.8-43.9) | 0.76 (0.47-1.25) | 0.78 (0.46-1.32) |
| Level of education |  |  |  | |  |  | |  |  |  |
| ≤12y | 728 (75.7%) | 727 (75.6%) | 64 (8.8%) | | 71 (9.8%) | 22.7 (17.1-28.3) | | 32.4 (24.9-39.9) | 0.76 (0.54-1.06) | 0.78 (0.54-1.13) |
| >12y | 226 (23.5%) | 226 (23.5%) | 11 (4.9%) | | 13 (5.8%) | 12.2 (5.0-19.5) | | 17.3 (7.9-26.6) | 0.78 (0.35-1.71) | 0.75 (0.32-1.78) |
| Type of statin drug at treatment start |  |  |  | |  |  | |  |  |  |
| Simvastatin | 329 (34.2%) | 329 (34.2%) | 33 (10.0%) | | 39 (11.9%) | 17.0 (11.2-22.8) | | 26.3 (18.1-34.6) | 0.71 (0.44-1.15) | 0.73 (0.44-1.20) |
| Atorvastatin | 589 (61.2%) | 589 (61.2%) | 40 (6.8%) | | 40 (6.8%) | 24.1 (16.7-31.6) | | 28.8 (19.9-37.7) | 0.87 (0.58-1.32) | 0.86 (0.53-1.40) |
| Other statins | 44 (4.6%) | 44 (4.6%) | 3 (6.8%) | | 5 (11.4%) | 21.1 (0.0-45.1) | | 46.9 (5.8-88.1) | 0.52 (0.12-2.29) | 0.60 (0.14-2.51) |

aHR, adjusted hazard ratio; CI, confidence interval; HR, hazard ratio; PY, person-years; y, years

*Propensity score matched for sex, age, level of education, year of diagnosis, disease duration, location and extent, healthcare utilization, and comorbidities

**Conditioned on matching set

**Table S5a** Incidence rates and hazard ratios of disease flares (systemic steroid use, start of immunomodulator or start/switch of anti-TNF treatment) in patients with ulcerative colitis with and without statin treatment 2006-2020

| **Outcome** | **N** | | **N events** | | | **Incidence rate (95% CI) per 1000 PY** | | **HR***  **(95%CI)** | **aHR****  **(95%CI)** |
| --- | --- | --- | --- | --- | --- | --- | --- | --- | --- |
|  | **Statin users** | **Non-statin users** | **Statin users** | **Non-statin users** | **Statin users** | | **Non-statin users** |  |  |
| **Overall** | 1 733 (100%) | 1 733 (100%) | 819 (47.3%) | 825 (47.6%) | 207.4 (193.2-221.6) | | 275.0 (256.2-293.8) | 0.85 (0.77-0.94) | 0.86 (0.77-0.97) |
| Follow-up time |  |  |  |  |  | |  |  |  |
| <1y | 1 733 (100%) | 1 733 (100%) | 511 (29.5%) | 597 (34.4%) | 409.0 (373.5-444.4) | | 526.5 (484.2-568.7) | 0.80 (0.71-0.90) | 0.81 (0.71-0.92) |
| 1-<5y | 984 (56.8%) | 821 (47.4%) | 263 (26.7%) | 192 (23.4%) | 124.8 (109.7-139.8) | | 122.6 (105.2-139.9) | 1.03 (0.85-1.25) | 1.11 (0.86-1.44) |
| ≥5y | 240 (13.8%) | 159 (9.2%) | 45 (18.8%) | 36 (22.6%) | 25.1 (17.8-32.4) | | 32.9 (22.1-43.6) | 0.65 (0.41-1.01) | 1.00 (0.38-2.66) |
| Sex |  |  |  |  |  | |  |  |  |
| Women | 782 (45.1%) | 782 (45.1%) | 371 (47.4%) | 376 (48.1%) | 219.2 (196.9-241.6) | | 285.5 (256.7-314.4) | 0.86 (0.75-1.00) | 0.90 (0.76-1.07) |
| Men | 951 (54.9%) | 951 (54.9%) | 448 (47.1%) | 449 (47.2%) | 198.4 (180.1-216.8) | | 266.8 (242.1-291.4) | 0.84 (0.73-0.96) | 0.84 (0.71-0.98) |
| Age |  |  |  |  |  | |  |  |  |
| 18-<60y | 590 (34.0%) | 637 (36.8%) | 292 (49.5%) | 311 (48.8%) | 194.5 (172.2-216.8) | | 246.2 (218.8-273.6) | 0.87 (0.74-1.02) | 0.98 (0.79-1.21) |
| ≥60y | 1 143 (66.0%) | 1 096 (63.2%) | 527 (46.1%) | 514 (46.9%) | 215.3 (196.9-233.7) | | 295.9 (270.3-321.5) | 0.84 (0.74-0.95) | 0.88 (0.75-1.02) |
| IBD diagnosis |  |  |  |  |  | |  |  |  |
| 2006-2010 | 749 (43.2%) | 749 (43.2%) | 391 (52.2%) | 388 (51.8%) | 170.2 (153.3-187.1) | | 251.5 (226.5-276.6) | 0.81 (0.70-0.93) | 0.85 (0.71-1.01) |
| 2011-2015 | 711 (41.0%) | 711 (41.0%) | 333 (46.8%) | 338 (47.5%) | 237.5 (212.0-263.0) | | 278.1 (248.5-307.8) | 0.89 (0.76-1.03) | 0.86 (0.72-1.03) |
| 2016-2019 | 273 (15.8%) | 273 (15.8%) | 95 (34.8%) | 99 (36.3%) | 380.2 (303.7-456.6) | | 408.9 (328.3-489.4) | 0.93 (0.70-1.24) | 0.94 (0.68-1.28) |
| Start year of follow-up |  |  |  |  |  | |  |  |  |
| 2006-2010 | 148 (8.5%) | 146 (8.4%) | 98 (66.2%) | 90 (61.6%) | 144.3 (115.7-172.9) | | 236.0 (187.3-284.8) | 0.78 (0.58-1.06) | 0.87 (0.61-1.25) |
| 2011-2015 | 535 (30.9%) | 537 (31.0%) | 337 (63.0%) | 315 (58.7%) | 197.4 (176.3-218.5) | | 237.1 (210.9-263.3) | 0.92 (0.79-1.07) | 0.93 (0.77-1.12) |
| 2016-2020 | 1 050 (60.6%) | 1 050 (60.6%) | 384 (36.6%) | 420 (40.0%) | 245.6 (221.1-270.2) | | 325.5 (294.4-356.6) | 0.81 (0.71-0.93) | 0.82 (0.70-0.96) |
| Level of education |  |  |  |  |  | |  |  |  |
| ≤12y | 1 346 (77.7%) | 1 349 (77.8%) | 649 (48.2%) | 641 (47.5%) | 205.4 (189.6-221.2) | | 271.7 (250.7-292.8) | 0.86 (0.77-0.96) | 0.85 (0.75-0.97) |
| >12y | 377 (21.8%) | 377 (21.8%) | 165 (43.8%) | 182 (48.3%) | 215.3 (182.4-248.2) | | 289.1 (247.1-331.2) | 0.81 (0.66-1.00) | 0.92 (0.72-1.18) |
| Type of statin drug at treatment start |  |  |  |  |  | |  |  |  |
| Simvastatin | 567 (32.7%) | 567 (32.7%) | 325 (57.3%) | 318 (56.1%) | 171.6 (153.0-190.3) | | 238.2 (212.0-264.4) | 0.84 (0.72-0.98) | 0.85 (0.70-1.03) |
| Atorvastatin | 1 080 (62.3%) | 1 080 (62.3%) | 466 (43.1%) | 469 (43.4%) | 237.5 (215.9-259.0) | | 295.2 (268.5-322.0) | 0.87 (0.77-0.99) | 0.88 (0.76-1.02) |
| Other statins | 86 (5.0%) | 86 (5.0%) | 28 (32.6%) | 38 (44.2%) | 298.6 (188.0-409.2) | | 497.2 (339.1-655.3) | 0.67 (0.42-1.08) | 0.76 (0.44-1.32) |

aHR, adjusted hazard ratio; CI, confidence interval; HR, hazard ratio; PY, person-years; y, years

*Propensity score matched for sex, age, level of education, year of diagnosis, disease duration, location and extent, healthcare utilization, and comorbidities

**Conditioned on matching set

**Table S5b** Incidence rates and hazard ratios of disease flares (systemic steroid use, start of immunomodulator or start/switch of anti-TNF treatment) in patients with Crohn’s disease with and without statin treatment 2006-2020

| **Outcome** | **N** | | | **N events** | | | **Incidence rate (95% CI) per 1000 PY** | | **HR***  **(95%CI)** | **aHR****  **(95%CI)** |
| --- | --- | --- | --- | --- | --- | --- | --- | --- | --- | --- |
|  | **Statin users** | **Non-statin users** | **Statin users** | | **Non-statin users** | **Statin users** | | **Non-statin users** |  |  |
| **Overall** | 962 (100%) | 962 (100%) | 496 (51.6%) | | 478 (49.7%) | 245.5 (223.9-267.1) | | 292.3 (266.1-318.5) | 0.96 (0.85-1.08) | 1.02 (0.88-1.19) |
| Follow-up time |  |  |  | |  |  | |  |  |  |
| <1y | 962 (100%) | 962 (100%) | 354 (36.8%) | | 355 (36.9%) | 551.3 (493.8-608.7) | | 568.6 (509.4-627.7) | 0.98 (0.84-1.13) | 1.04 (0.88-1.23) |
| 1-<5y | 475 (49.4%) | 448 (46.6%) | 126 (26.5%) | | 112 (25.0%) | 119.7 (98.8-140.6) | | 133.6 (108.9-158.4) | 0.92 (0.72-1.18) | 0.89 (0.60-1.31) |
| ≥5y | 133 (13.8%) | 81 (8.4%) | 16 (12.0%) | | 11 (13.6%) | 16.2 (8.2-24.1) | | 19.0 (7.8-30.3) | 0.80 (0.38-1.68) | 2.00 (0.37-10.92) |
| Sex |  |  |  | |  |  | |  |  |  |
| Women | 486 (50.5%) | 486 (50.5%) | 254 (52.3%) | | 236 (48.6%) | 262.6 (230.3-294.9) | | 281.2 (245.3-317.1) | 1.05 (0.88-1.24) | 1.11 (0.89-1.37) |
| Men | 476 (49.5%) | 476 (49.5%) | 242 (50.8%) | | 242 (50.8%) | 229.8 (200.9-258.8) | | 304.0 (265.7-342.3) | 0.88 (0.74-1.05) | 0.94 (0.76-1.17) |
| Age |  |  |  | |  |  | |  |  |  |
| 18-<60y | 377 (39.2%) | 384 (39.9%) | 188 (49.9%) | | 174 (45.3%) | 214.0 (183.4-244.6) | | 235.6 (200.6-270.6) | 1.01 (0.83-1.23) | 1.08 (0.81-1.43) |
| ≥60y | 585 (60.8%) | 578 (60.1%) | 308 (52.6%) | | 304 (52.6%) | 269.7 (239.6-299.9) | | 339.1 (300.9-377.2) | 0.93 (0.79-1.09) | 0.95 (0.77-1.16) |
| IBD diagnosis |  |  |  | |  |  | |  |  |  |
| 2006-2010 | 451 (46.9%) | 451 (46.9%) | 246 (54.5%) | | 233 (51.7%) | 209.7 (183.5-235.9) | | 246.2 (214.6-277.8) | 0.97 (0.82-1.14) | 1.05 (0.84-1.31) |
| 2011-2015 | 367 (38.1%) | 367 (38.1%) | 178 (48.5%) | | 182 (49.6%) | 245.7 (209.6-281.8) | | 319.0 (272.7-365.4) | 0.88 (0.72-1.08) | 0.91 (0.71-1.16) |
| 2016-2019 | 144 (15.0%) | 144 (15.0%) | 72 (50.0%) | | 63 (43.8%) | 587.3 (451.7-723.0) | | 532.8 (401.2-664.3) | 1.19 (0.85-1.66) | 1.25 (0.84-1.86) |
| Start year of follow-up |  |  |  | |  |  | |  |  |  |
| 2006-2010 | 80 (8.3%) | 80 (8.3%) | 56 (70.0%) | | 56 (70.0%) | 193.9 (143.1-244.7) | | 242.2 (178.8-305.7) | 0.90 (0.66-1.22) | 0.97 (0.60-1.58) |
| 2011-2015 | 313 (32.5%) | 313 (32.5%) | 194 (62.0%) | | 188 (60.1%) | 194.9 (167.5-222.4) | | 256.4 (219.8-293.1) | 0.91 (0.75-1.11) | 1.01 (0.79-1.29) |
| 2016-2020 | 569 (59.1%) | 569 (59.1%) | 246 (43.2%) | | 234 (41.1%) | 334.1 (292.4-375.9) | | 348.8 (304.1-393.5) | 1.01 (0.84-1.20) | 1.04 (0.85-1.28) |
| Level of education |  |  |  | |  |  | |  |  |  |
| ≤12y | 728 (75.7%) | 727 (75.6%) | 386 (53.0%) | | 369 (50.8%) | 257.3 (231.6-282.9) | | 302.0 (271.2-332.9) | 0.97 (0.85-1.11) | 1.02 (0.86-1.21) |
| >12y | 226 (23.5%) | 226 (23.5%) | 105 (46.5%) | | 105 (46.5%) | 207.0 (167.4-246.7) | | 260.1 (210.4-309.9) | 0.91 (0.70-1.18) | 1.01 (0.74-1.39) |
| Type of statin drug at treatment start |  |  |  | |  |  | |  |  |  |
| Simvastatin | 329 (34.2%) | 329 (34.2%) | 196 (59.6%) | | 192 (58.4%) | 212.2 (182.5-241.9) | | 262.7 (225.6-299.9) | 0.94 (0.79-1.13) | 1.06 (0.83-1.36) |
| Atorvastatin | 589 (61.2%) | 589 (61.2%) | 282 (47.9%) | | 273 (46.3%) | 283.5 (250.4-316.6) | | 332.7 (293.2-372.2) | 0.94 (0.80-1.11) | 0.98 (0.81-1.19) |
| Other statins | 44 (4.6%) | 44 (4.6%) | 18 (40.9%) | | 13 (29.5%) | 176.5 (95.0-258.1) | | 155.1 (70.8-239.4) | 1.48 (0.76-2.91) | 1.40 (0.62-3.15) |

aHR, adjusted hazard ratio; CI, confidence interval; HR, hazard ratio; PY, person-years; y, years

*Propensity score matched for sex, age, level of education, year of diagnosis, disease duration, location and extent, healthcare utilization, and comorbidities

**Conditioned on matching set

**References**

1. Ludvigsson JF, Almqvist C, Bonamy AK, et al. Registers of the Swedish total population and their use in medical research. Eur J Epidemiol 2016;31:125-36.

2. Ludvigsson JF, Andersson E, Ekbom A, et al. External review and validation of the Swedish national inpatient register. BMC Public Health 2011;11:450.

3. Forss A, Myrelid P, Olen O, et al. Validating surgical procedure codes for inflammatory bowel disease in the Swedish National Patient Register. BMC Med Inform Decis Mak 2019;19:217.

4. Ludvigsson JF, Otterblad-Olausson P, Pettersson BU, et al. The Swedish personal identity number: possibilities and pitfalls in healthcare and medical research. Eur J Epidemiol 2009;24:659-67.

5. Ludvigsson JF, Svedberg P, Olen O, et al. The longitudinal integrated database for health insurance and labour market studies (LISA) and its use in medical research. Eur J Epidemiol 2019;34:423-437.

6. Wettermark B, Hammar N, Fored CM, et al. The new Swedish Prescribed Drug Register--opportunities for pharmacoepidemiological research and experience from the first six months. Pharmacoepidemiol Drug Saf 2007;16:726-35.

7. Ludvigsson JF, Andersson M, Bengtsson J, et al. Swedish Inflammatory Bowel Disease Register (SWIBREG) - a nationwide quality register. Scandinavian journal of gastroenterology. 2019;54:1089-1101.
